# Eicosanoid regulation of debris-stimulated metastasis

**DOI:** 10.1073/pnas.2107771118

**Published:** 2021-10-04

**Authors:** Jianjun Deng, Haixia Yang, Victoria M. Haak, Jun Yang, Franciele C. Kipper, Chantal Barksdale, Sung Hee Hwang, Allison Gartung, Diane R. Bielenberg, Selvakumar Subbian, Koc-Kan Ho, Xiang Ye, Daidi Fan, Yongkui Sun, Bruce D. Hammock, Dipak Panigrahy

**Affiliations:** ^a^Center for Vascular Biology Research, Beth Israel Deaconess Medical Center, Harvard Medical School, Boston, MA 02215;; ^b^Department of Pathology, Beth Israel Deaconess Medical Center, Harvard Medical School, Boston, MA 02215;; ^c^Cancer Center, Beth Israel Deaconess Medical Center, Harvard Medical School, Boston, MA 02215;; ^d^Shaanxi Key Laboratory of Degradable Biomedical Materials, School of Chemical Engineering, Northwest University, Xi'an 710069, China;; ^e^College of Food Science and Nutritional Engineering, China Agricultural University, Beijing 100083, China;; ^f^Department of Entomology and Nematology, University of California, Davis, CA 95616;; ^g^UCD Comprehensive Cancer Center, University of California, Davis, CA 95616;; ^h^Vascular Biology Program, Boston Children's Hospital, Harvard Medical School, Boston, MA 02115;; ^i^Public Health Research Institute, New Jersey Medical School, Rutgers University, Newark, NJ07103;; ^j^Shenzhen Bay Laboratory, Gaoke Innovation Center, Shenzhen 518000, China;; ^k^Institute of Biopharmaceutical and Health Engineering, Tsinghua Shenzhen International Graduate School, Tsinghua University, Shenzhen 518055, China;; ^l^Division of Discovery Research, Ionova Life Science Co., Ltd., Shenzhen 518118, China;; ^m^Division of Discovery Research, Ionova Biotherapeutics Co., Inc., Foshan 528000, China

**Keywords:** debris, autacoid, soluble epoxide hydrolase, prostaglandin E_2_ receptor 4, inflammation resolution

## Abstract

Cancer therapy reduces tumor burden via tumor cell death (“debris”), which can accelerate tumor progression via the failure of inflammation resolution. Thus, there is an urgent need to develop treatment modalities that stimulate the clearance or resolution of inflammation-associated debris. Here, we demonstrate that chemotherapy-generated debris stimulates metastasis by up-regulating soluble epoxide hydrolase (sEH) and the prostaglandin E_2_ receptor 4 (EP4). Therapy-induced tumor cell debris triggers a storm of proinflammatory and proangiogenic eicosanoid-driven cytokines. Thus, targeting a single eicosanoid or cytokine is unlikely to prevent chemotherapy-induced metastasis. Pharmacological abrogation of both sEH and EP4 eicosanoid pathways prevents hepato-pancreatic tumor growth and liver metastasis by promoting macrophage phagocytosis of debris and counterregulating a protumorigenic eicosanoid and cytokine storm. Therefore, stimulating the clearance of tumor cell debris via combined sEH and EP4 inhibition is an approach to prevent debris-stimulated metastasis and tumor growth.

## Significance

Cancer therapy, such as chemotherapy, induces tumor cell death (“debris”), which can stimulate metastasis. Chemotherapy-generated debris upregulates soluble epoxide hydrolase (sEH) and the prostaglandin E_2_ receptor 4 (EP4), which triggers a macrophage-derived storm of proinflammatory and proangiogenic lipid autacoid and cytokine mediators. Although sEH inhibitors and EP4 antagonists are in clinical development for multiple inflammatory diseases, their combined role in cancer is unknown. Here, we show that the synergistic antitumor activity of sEH and EP4 inhibition suppresses hepato-pancreatic tumor growth, without overt toxicity, via macrophage phagocytosis of debris and counterregulation of a debris-stimulated cytokine storm. Thus, stimulating the resolution of inflammation via combined inhibition of sEH and EP4 may be an approach for preventing metastatic progression driven by cancer therapy.

Hepatocellular carcinoma (HCC) is a leading cause of cancer death and the most rapidly increasing cancer in the United States (1). Pancreatic cancer is the fourth leading cause of cancer-related deaths (2). Both of these cancer types are associated with a poor prognosis (1, 2). Despite the effectiveness of chemotherapy as a frontline cancer treatment, accumulating evidence from animal models suggests that chemotherapy may stimulate tumor growth and metastasis (3–22). The Révész effect, described in 1956, demonstrates that tumor cell death (“debris”) generated by cancer therapy, such as radiation, accelerates tumor engraftment (23). Follow-up studies have confirmed the Révész effect, whereby radiation-generated debris stimulates tumor growth via a proinflammatory response (24–29). Dead cell–derived mediators also stimulate tumor cell growth (30, 31). Notably, large numbers of cells are known to die in established tumors (32), which can lead to endogenous tumor-promoting debris in the tumor microenvironment (8, 33–35).

Chemotherapy-generated tumor cell debris (e.g., apoptotic and necrotic cells) promotes tumor growth and metastasis via several mechanisms, including: 1) triggering a storm of proinflammatory and proangiogenic eicosanoids and cytokines (8, 9, 33, 35–38); 2) hijacking tumor-associated macrophages (TAMs) (37, 39); 3) inactivating M1-like TAMs (37); and 4) inducing immunosuppression and limiting antitumor immunity (40–42). Importantly, a metastatic phenotype and poor survival in cancer patients can be predicted by high levels of tumor cell debris (43–48). Thus, every attempt to induce tumor cell death is a double-edged sword as the resulting debris stimulates the growth of surviving tumor cells (8, 25, 33, 34, 35, 37, 38, 49–53). Tumor cells that survive treatment with chemotherapy or radiation undergo tumor cell repopulation (29). Yet, no strategy currently exists to stimulate the clearance or resolution of therapy-induced tumor cell debris and inflammation in cancer patients (35, 54).

The failure to resolve inflammation-associated debris critically drives the pathogenesis of many human diseases, including cancer (8, 35, 55). Inflammation is regulated by a balance between inflammation-initiating eicosanoids (e.g., prostaglandins, leukotrienes, and thromboxanes) and specialized proresolving lipid autacoid mediators (SPMs; e.g., resolvins and lipoxins), which are endogenously produced in multiple tissues throughout the human body (56). Notably, arachidonic acid metabolites, collectively called eicosanoids, are potent mediators of inflammation and cancer metastasis (57, 58). Epoxyeicosatrienoic acids (EETs, also named EpETrEs), key eicosanoid regulators of angiogenesis, also stimulate inflammation resolution via macrophage-mediated phagocytosis of cell debris (59–64). Because EETs are rapidly metabolized by soluble epoxide hydrolase (sEH) to the less active dihydroxyeicosatrienoic acids (DiHETEs) (62), inhibition of sEH stabilizes EETs (62, 65). Indeed, sEH is a key therapeutic target for pain, as well as neurodegenerative and inflammatory diseases, including cancer (33, 35, 65–74). Thus, sEH regulates inflammatory responses (62). Importantly, sEH inhibition reduces the circulating levels and the expression of pancreatic mRNA of inflammatory cytokines, including tumor necrosis factor (TNF)-α, interleukin (IL)-1β, and IL-6 in experimental acute pancreatitis in mice (75). Chronic pancreatitis is essential for the induction of pancreatic ductal adenocarcinoma by K-Ras oncogenes in adult mice, suggesting that inflammation is a critical driver of pancreatic cancer (76, 77). Potent, selective inhibitors of sEH have been demonstrated to suppress human cancers (e.g., glioblastoma) and inflammation-induced carcinogenesis (67, 71). Similarly, inhibition of sEH can suppress inflammatory bowel disease-induced carcinogenesis and inflammation-associated pancreatic cancer (74, 78). In addition, a dual inhibitor of c-RAF and sEH suppresses chronic pancreatitis and murine pancreatic intraepithelial neoplasia in mutant K-Ras–initiated carcinogenesis (72, 73). Likewise, dual cyclooxygenase-2 (COX-2)/sEH inhibitors (e.g., PTUPB) potentiate the antitumor activity of chemotherapy and suppress primary tumor growth and metastasis via inflammation resolution (33, 35, 66, 70).

Cancer therapy-induced debris can stimulate tumor growth and metastasis via prostaglandin E_2_ (PGE_2_) in the tumor microenvironment (25, 35, 79). PGE_2_ exerts its biological activity via four G protein-coupled receptors: EP1, EP2, EP3, and EP4 (80). Among these, EP4 is upregulated in both tumor cells and immune cells (e.g., macrophages) and exhibits protumorigenic activity in many human malignancies (e.g., breast, prostate, colon, ovarian, and lung) by regulating angiogenesis, lymphangiogenesis, liver metastasis, and lymphatic metastasis (81–85). Interestingly, PGE_2_ impairs macrophage phagocytosis of pathogens via EP4 receptor activation (86–88). Moreover, EP4 stimulates cancer proliferation, migration, invasion, and metastasis (89). EP4 gene silencing inhibits metastatic potential in vivo in preclinical models of breast, prostate, colon, and lung cancer (85, 90). Additionally, EP4 antagonists can suppress proinflammatory cytokines (e.g., C-C motif chemokine ligand 2 [CCL2], IL-6, and C-X-C chemokine motif 8 [CXCL8]), reduce inflammation-dependent bone metastasis, and diminish immunosuppression, while restoring antitumor immunity (91–93). In a clinical study, the EP4 antagonist E7046 increased the levels of T cells and tumor-infiltrating M2 macrophages in patients with advanced malignancies (94). Intriguingly, EP4 antagonists enhance the tumor response to chemotherapy by inducing extracellular vesicle-mediated clearance of cancer cells (95). Notably, EP4 antagonists reverse chemotherapy resistance or enhance immune-based therapies in various tumor types, including lymphoma, colorectal cancer, and lung cancer (80, 93, 96). Thus, targeting the EP4 receptor may be a strategy to suppress debris-stimulated tumor growth and metastasis.

Here, we demonstrate that tumor cell debris generated by chemotherapy (e.g., gemcitabine) stimulates primary hepato-pancreatic cancer growth and metastasis when coinjected with a subthreshold (nontumorigenic) inoculum of tumor cells. Chemotherapy-generated debris upregulated sEH and EP4, which triggered a macrophage-derived storm of proinflammatory and proangiogenic mediators. Inhibitors of sEH and EP4 antagonists promoted inflammation resolution through macrophage phagocytosis of tumor cell debris and reduced proinflammatory eicosanoid and cytokine production in the tumor microenvironment. Altogether, our data show that the combined pharmacological abrogation of sEH and EP4 can prevent hepato-pancreatic cancer and metastatic progression.

## Results

### Chemotherapy-Generated Tumor Cell Debris Stimulates Pancreatic Cancer via Upregulation of sEH and EP4.

**Fig. 1.**
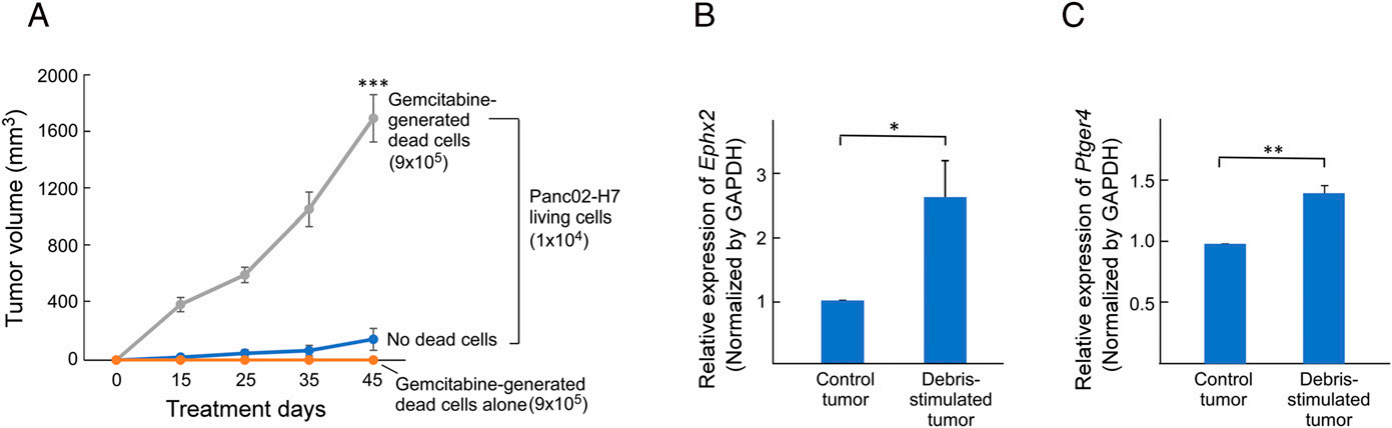
Chemotherapy-generated tumor cell debris stimulates pancreatic cancer via upregulation of sEH and EP4. (*A*) Pancreatic tumor growth stimulated by gemcitabine-generated Panc02-H7 debris (9 × 10^5^ dead cells) coinjected with a subthreshold inoculum of Panc02-H7 (1 × 10^4^ living cells). *n* = 5 mice per group. ***P < 0.001 vs. dead cells or living cells (“no dead cells”) alone. Relative gene expression of (*B*) *Ephx2* (sEH) and (*C*) *Ptger4* (EP4) in debris-stimulated tumor tissue (9 × 10^5^ gemcitabine-generated Panc02-H7 dead cells + 1 × 10^4^ Panc02-H7 living cells) vs. control tumor (1 × 10^4^ Panc-02-H7 living cells). mRNA expression levels of genes were analyzed by qRT-PCR and normalized by GAPDH. *n* = 3 per group. *P < 0.05, **P < 0.01 vs. control.

To investigate whether chemotherapy-generated debris is biologically relevant in pancreatic cancer, we first developed a debris-stimulated pancreatic adenocarcinoma model. Gemcitabine, a first-line chemotherapy for pancreatic cancer, induced cancer cell death, generating debris (e.g., apoptotic/necrotic cells and cell fragments) in pancreatic tumor cell lines (8). Injection of a subthreshold inoculum of living tumor cells (e.g., 10^4^ cells) models tumor dormancy (8, 42). Gemcitabine-generated pancreatic adenocarcinoma (Panc02-H7) cellular debris (9 × 10^5^ dead cells) coinjected with Panc02-H7 (10^4^ living cells) stimulated primary tumor growth in immunocompetent C57BL/6J mice compared to living cells alone (Fig. 1*A*). In contrast, mice injected with gemcitabine-generated debris alone (with no living cells) did not exhibit tumor growth, even at 45 d postinjection (Fig. 1*A*). Since sEH promotes inflammation and subsequent carcinogenesis (35, 72, 73, 78), we measured the expression levels of sEH in debris-stimulated (combination of dead cells and living cells) versus control (living cells only) tumors. Gene-expression levels of *Ephx2*, which encodes murine sEH, were increased in gemcitabine-induced debris-stimulated Panc02-H7 tumors compared to size-matched Panc02-H7 tumors derived from living tumor cells (Fig. 1*B*). Since the biological activity of PGE_2_ is mediated by four receptors (EP1, EP2, EP3, and EP4), we next investigated whether the PGE_2_ receptors were upregulated in debris-stimulated pancreatic tumors. *Ptger4*, which encodes murine EP4, was upregulated in debris-stimulated Panc02-H7 tumors compared to Panc02-H7 tumors derived from only living tumor cells (with no debris) (Fig. 1*C*). In contrast, there were no significant differences in the expression of *Ptger1*, *Ptger2*, or *Ptger3* (which encode EP1, EP2, and EP3, respectively) in the debris-stimulated Panc02-H7 tumors compared to control (*SI Appendix*, Fig. S1). Thus, chemotherapy-generated pancreatic tumor cell debris stimulates tumor growth via upregulated sEH and EP4 expression.

### Combined Inhibition of sEH and EP4 Prevents a Debris-Stimulated Macrophage-Derived Cytokine Storm.

**Fig. 2.**
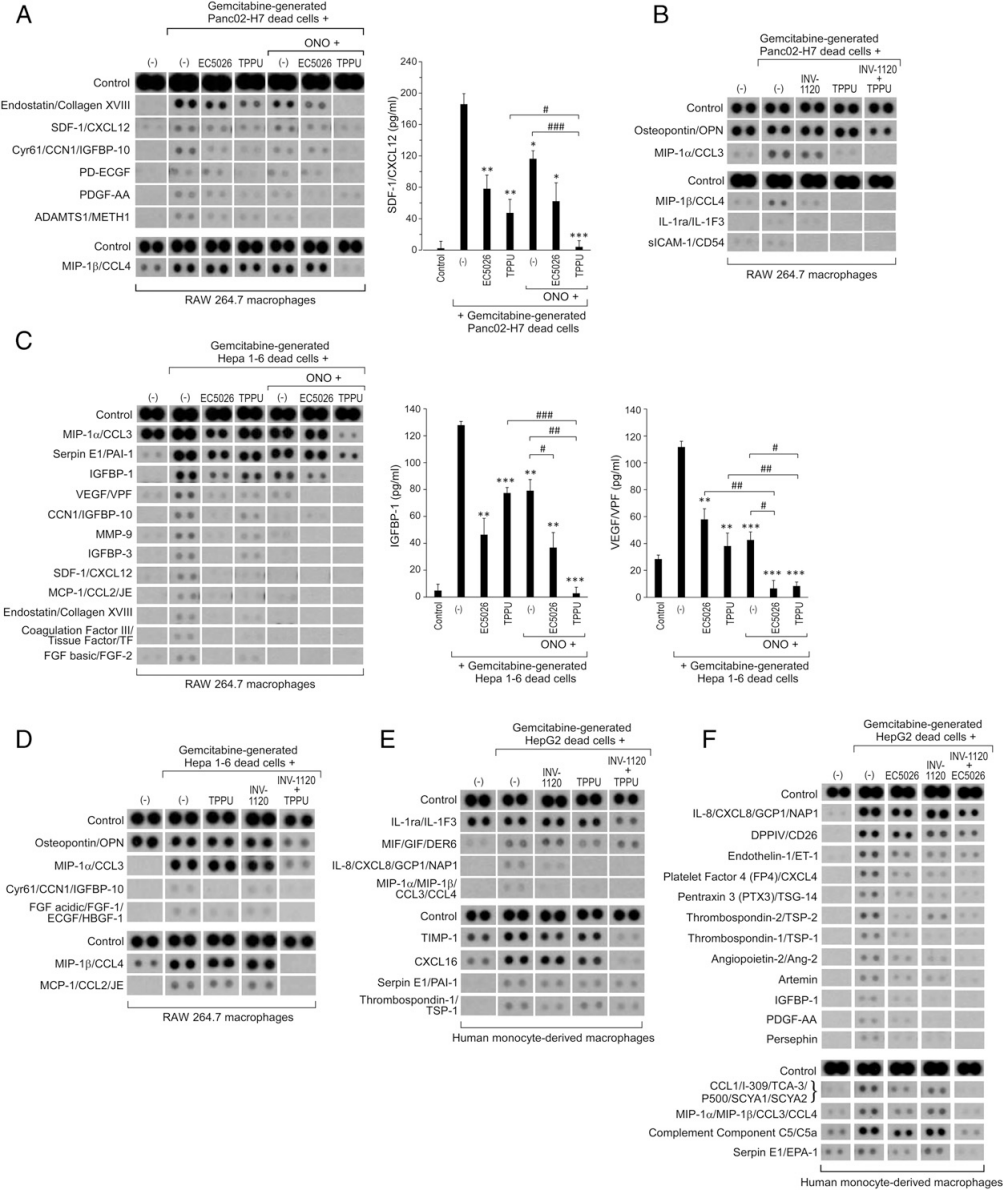
The cytokine storm triggered by debris-stimulated macrophages is prevented by combined sEH and EP4 inhibition. (*A* and *B*) Angiogenic (*Upper*) and inflammatory (*Lower*) cytokines from conditioned medium of RAW 264.7 macrophages treated with vehicle, sEH inhibitor (EC5026 or TPPU, 10 µM, 2 h), EP4 antagonist (INV-1120 or ONO-AE3-208, 10 µM, 2 h), or a combination (EC5026 + ONO-AE3-208, TPPU + ONO-AE3-208, or TPPU + INV-1120, 10 µM each, 2 h) and subsequently stimulated by gemcitabine-generated Panc02-H7 tumor cell debris vs. no debris. The SDF-1/CXCL12 released from RAW 264.7 macrophages was quantified by ELISA. Data are presented as means (pg/mL) ± SEM *n* = 3 per group. *P < 0.05, **P < 0.01, ***P < 0.001 vs. dead cells + Panc02-H7 living cells. ^#^*P* < 0.05, ^###^*P* < 0.001 vs. TPPU + ONO-AE3-208. (*C* and *D*) Angiogenic (*Upper*) and inflammatory (*Lower*) cytokines from conditioned medium of RAW 264.7 macrophages treated with vehicle, sEH inhibitor (EC5026 or TPPU, 10 µM, 2 h), EP4 antagonist (INV-1120 or ONO-AE3-208, 10 µM, 2 h), or a combination (EC5026 + ONO-AE3-208, TPPU + ONO-AE3-208, or TPPU + INV-1120, 10 µM each, 2 h) and subsequently stimulated by gemcitabine-generated Hepa 1-6 tumor cell debris vs. no debris. The IGFBP-1 and VEGF/VPF released from RAW 264.7 macrophages were quantified by ELISA. Data are presented as means (pg/mL) ± SEM *n* = 3 per group. **P < 0.01, ***P < 0.001 vs. dead cells + Hepa 1-6 living cells. ^#^*P* < 0.05, ^##^*P* < 0.01, ^###^*P* < 0.001. (*E* and *F*) Angiogenic (*Upper*) and inflammatory (*Lower*) cytokines from conditioned medium of hMDMs treated with vehicle, sEH inhibitor (EC5026 or TPPU, 10 µM, 2 h), EP4 antagonist (INV-1120, 10 µM, 2 h), or a combination (EC5026 + INV-1120 or TPPU + INV-1120, 10 µM each, 2 h) and subsequently stimulated by gemcitabine-generated HepG2 tumor cell debris vs. no debris.

Debris generated by cancer therapy (e.g., chemotherapy or radiation) can trigger a macrophage-derived storm of protumorigenic cytokines (8, 33, 35, 97). Thus, we assessed the release of cytokines by RAW 264.7 macrophages cocultured with gemcitabine-generated Panc02-H7 debris. Indeed, debris-stimulated macrophages triggered seven proinflammatory and proangiogenic mediators—including stromal cell–derived factor (SDF)/CXCL12, cysteine-rich angiogenic protein 61 (Cyr61)/cellular communication network factor (CCN)1 insulin like growth factor binding protein (IGFBP)-10, platelet-derived endothelial cell growth factor (PD-ECGF), platelet-derived growth factor AA (PDGF-AA), ADAM metalloproteinase with thrombospondin type 1 motif 1 (ADAMTS1)/metalloproteinase and thrombospondin domains (METH)1, macrophage inflammatory protein (MIP)-1α/C-C motif chemokine ligand (CCL)3, and MIP-1β/CCL4—compared to macrophages not exposed to debris (Fig. 2 *A* and *B*). Macrophages stimulated by gemcitabine-generated Panc02-H7 debris also secreted antiangiogenic mediators including endostatin/collagen XVIII (Fig. 2*A*). To confirm that the debris-stimulated cytokine storm was not specific to pancreatic cancer, we next utilized a liver cancer cell line: Hepa 1-6 hepatoma (murine hepatocellular carcinoma cell line). Similarly, gemcitabine-generated Hepa 1-6 debris triggered 12 proinflammatory and proangiogenic cytokine mediators, including Serpin E1/ plasminogen activator inhibitor 1 (PAI-1), IGFBP-1, vascular endothelial growth factor (VEGF)/ vascular permeability factor (VPF), CCN1/IGFBP-10, matrix metalloproteinase (MMP)-9, IGFBP-3, SDF-1/CXCL12, monocyte chemoattractant protein-1 (MCP-1)/CCL2/JE, coagulation factor III/tissue factor (TF), basic fibroblast growth factor (FGF)/FGF-2, MIP-1α/CCL3, and MIP-1β/CCL4 from RAW 264.7 macrophages (Fig. 2 *C* and *D*).

To exclude that the debris-stimulated cytokine storm was specific to RAW 264.7 macrophages, we next conducted cytokine array screening of conditioned medium from primary human monocyte-derived macrophages (hMDM) stimulated with gemcitabine-generated HepG2 (human hepatocellular carcinoma cell line) debris. Indeed, gemcitabine-generated HepG2 debris also triggered a proinflammatory cytokine storm by hMDMs, including IL-8/CXCL8/ granulocyte chemotactic protein 1 (GCP1)/nucleosome assembly protein 1 (NAP1), MIP-1α/MIP-1β/CCL3/CCL4, Serpin E1/PAI-1, CCL1/inflammatory cytokine 309 (I-309)/thymus-derived chemotactic agent (TCA)-3/C-C motif chemokine 1 (P500)/ small inducible cytokine A1 (SCYA1)/SCYA2, dipeptidyl peptidase 4 (DPPIV)/cluster of differentiation (CD)26, endothelin-1 (ET-1), Pentraxin 3 (PTX3)/TNF-stimulated gene-14 (TSG-14), angiopoietin-2 (Ang-2), Artemin, IGFBP-1, PDGF-AA, and Persephin (Fig. 2 *E* and *F*). The hMDMs stimulated by gemcitabine-generated HepG2 debris also secreted antiinflammatory and antiangiogenic mediators, including thrombospondin-1 (TSP-1), TSP-2, and platelet factor 4 (PF4)/CXCL4, in contrast to macrophages not exposed to the debris (Fig. 2 *E* and *F*).

We next asked whether combined pharmacological inhibition of sEH and EP4 could suppress the debris-stimulated cytokine storm, given that individual cytokine blockade does not prevent debris-stimulated tumor growth (8). RAW 264.7 macrophages or hMDMs were treated with various concentrations of an sEH inhibitor (TPPU or EC5026) and an EP4 antagonist (INV-1120 or ONO-AE3-208) prior to stimulating with gemcitabine-generated Panc02-H7 debris. Neither the sEH inhibitor nor the EP4 antagonist prevented the cytokine storm released by macrophages stimulated with gemcitabine-generated Panc02-H7 debris (*SI Appendix*, Fig. S2). In contrast, the combination treatment with the sEH inhibitor TPPU (10 µM) and the EP4 antagonist ONO-AE3-208 (10 µM) suppressed the gemcitabine-induced Panc02-H7 debris-stimulated RAW 264.7 macrophage-derived cytokine storm, including SDF-1/CXCL12, Cyr61/CCN1/IGFBP-10, PD-ECGF, PDGF-AA, ADAMTS1/METH1, MIP-1α/CCL3, and MIP-1β/CCL4, compared with either TPPU (10 µM) alone or ONO-AE3-208 (10 µM) alone (Fig. 2 *A* and *B*). The combination treatment with TPPU and ONO-AE3-208 (10 µM each) also prevented the cytokine storm released from RAW 264.7 macrophages stimulated by gemcitabine-generated Hepa 1-6 debris, including MIP-1α/CCL3, Serpin E1/PAI-1, IGFBP-1, VEGF/VPF, CCN1/IGFBP-10, MMP-9, IGFBP-3, SDF-1/CXCL12, MCP-1/CCL2/JE, coagulation factor III/TF, and basic FGF/FGF-2, as compared to treatment with TPPU (10 µM) alone or ONO-AE3-208 (10 µM) alone (Fig. 2*C*). Moreover, the combination of TPPU and INV-1120 (10 µM each) also suppressed osteopontin (OPN), MIP-1α/CCL3, SDF-1/CXCL12, and MIP-1β/CCL4 secretion by RAW 264.7 macrophages stimulated with gemcitabine-generated Panc02-H7 debris (Fig. 2*B*) and suppressed OPN, MIP-1α/CCL3, Cyr61/CCN1/IGFBP-10, acidic FGF/FGF-1/ECGF/HBGF-1, MIP-1β/CCL4, and MCP-1/CCL2/JE production by RAW 264.7 macrophages stimulated with gemcitabine-generated Hepa 1-6 debris (Fig. 2*D*).

Additionally, the combination of TPPU and INV-1120 (10 µM each) inhibited the release of IL-8/CXCL8/GCP1/NAP1, tissue inhibitor of metalloproteinase 1 (TIMP-1), and CXCL16 by primary hMDMs stimulated with gemcitabine-generated HepG2 debris (Fig. 2*E*). The sEH inhibitor EC5026 (10 µM) inhibited the gemcitabine-induced Hepa 1-6 debris-stimulated macrophage-derived cytokine storm (Fig. 2*C*). In addition, the combination of EC5026 and INV-1120 (10 µM each) suppressed the production of PF4/CXCL4, PTX3/TSG-14, TSP-2, TSP-1, Ang-2, Artemin, IGFBP-1, PDGF-AA, CCL1/I-309/TCA-3/P500/SCYA1/SCYA2, MIP-1α/MIP-1β/CCL3/CCL4, complement component C5/C5a, and Serpin E1/PAI-1 by primary hMDMs stimulated with gemcitabine-generated HepG2 debris (Fig. 2*F*). VEGF/VPF, MCP1/CCL2/JE, basic FGF/FGF-2, and Serpin E1/PAI-1 each exhibit potent protumorigenic activity via proangiogenic and proinflammatory mechanisms (98–101). Therefore, the combined pharmacologic inhibition of sEH and EP4 inhibits the cytokine storm released by chemotherapy-induced debris-stimulated macrophages.

### Combined Inhibition of sEH and EP4 Stimulates Macrophage Phagocytosis of Debris via Suppression of NF-κB Signaling.

**Fig. 3.**
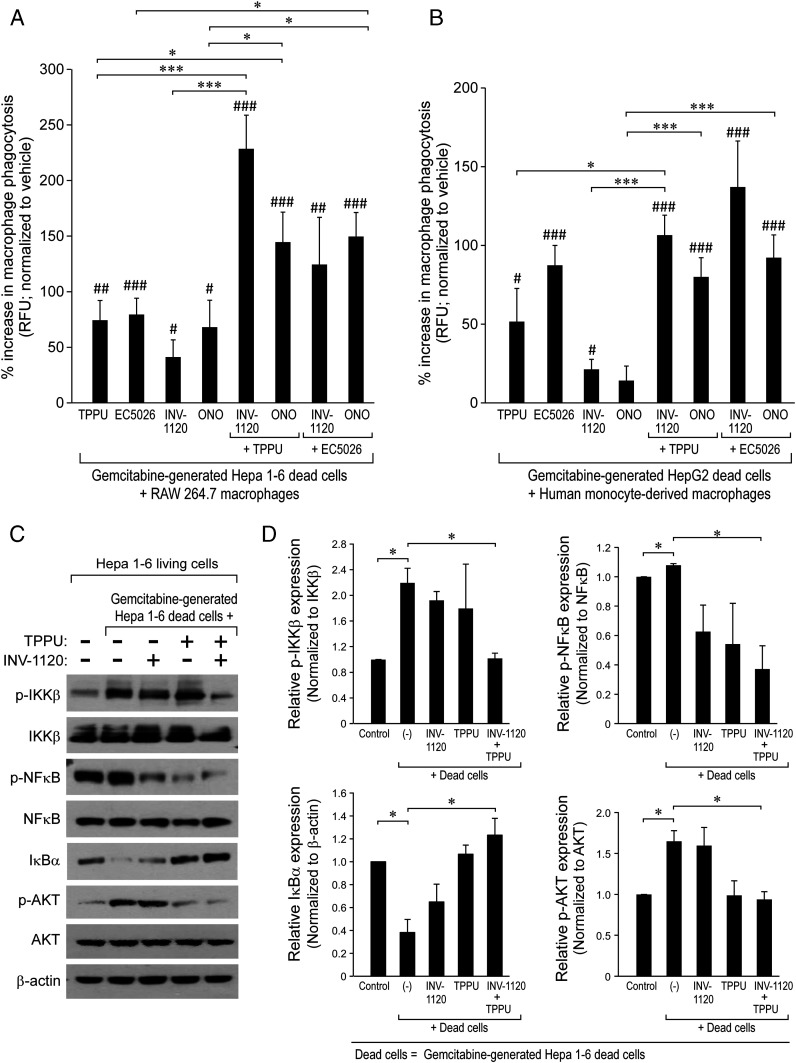
Combined inhibition of sEH and EP4 stimulates macrophage phagocytosis of debris via suppression of NF-κB signaling. Combination treatment with an sEH inhibitor (EC5026 or TPPU, 10 µM) and an EP4 antagonist (INV-1120 or ONO-AE3-208, 10 µM) for 2 h stimulates (*A*) RAW 264.7 murine macrophage phagocytosis or (*B*) hMDM phagocytosis of CFDA-labeled gemcitabine-generated Hepa 1-6 or HepG2 tumor cell debris. Macrophage phagocytosis was measured as RFU and normalized to percent increase above vehicle-treated macrophages. *n* = 12 per group. *P < 0.05, ***P < 0.001; ^#^*P* < 0.05, ^##^*P* < 0.01, ^###^*P* < 0.001 vs. vehicle. (*C*) Western blot analysis of p-IKKβ, IKKβ, p-NF-κB, NF-κB, IκBα, p-AKT, and AKT in living or debris-stimulated Hepa 1-6 tumors from mice treated for 28 d with vehicle, INV-1120 (5 mg/kg/d), TPPU (5 mg/kg/d), or INV-1120 + TPPU (5 mg/kg/d each). β-Actin was used as a loading control. (*D*) Quantification of protein expression shown in *C*. *n* = 3 mice per group. *P < 0.05.

A critical function of the resolution of inflammation is the clearance of debris via nonphlogistic macrophage phagocytosis (55). To this end, we assessed macrophage phagocytosis of chemotherapy-generated tumor cell debris in response to inhibition of sEH and EP4. Notably, combined inhibition of sEH and EP4 stimulated RAW 264.7 or hMDM phagocytosis of gemcitabine-generated Hepa 1-6 or HepG2 tumor cell debris, respectively, to a greater extent than the sEH inhibitors or EP4 antagonists alone (Fig. 3 *A* and *B*). Since NF-κB plays a critical role in inflammation and tumorigenesis (102), we next determined the expression of NF-κB in the debris-stimulated tumor models. Phosphorylation of IKKβ and NF-κB was increased and IκBα expression was decreased in debris-stimulated Hepa 1-6 tumors compared to Hepa 1-6 tumors derived from only living tumor cells (with no debris) (Fig. 3 *C* and *D*). In contrast, combined treatment with TPPU and INV-1120 reduced debris-stimulated phosphorylation of IKKβ and NF-κB and reversed debris-suppressed IκBα expression (Fig. 3 *C* and *D*). Similarly, gemcitabine-generated Hepa 1-6 or HepG2 tumor cell debris increased phosphorylation of IKKβ and NF-κB in RAW 264.7 macrophages or hMDMs, respectively (*SI Appendix*, Fig. S3). Moreover, the combination of TPPU and INV-1120 dramatically reduced the AKT phosphorylation in gemcitabine-generated debris-stimulated Hepa 1-6 tumors (Fig. 3 *C* and *D*). Thus, debris-stimulated tumor growth can be mediated by proinflammatory NF-κB signaling, which can be counterregulated by sEH and EP4 inhibition.

### Debris-Stimulated Macrophages Trigger Eicosanoids, Which Can Be Modulated by Combined Inhibition of sEH and EP4.

**Fig. 4.**
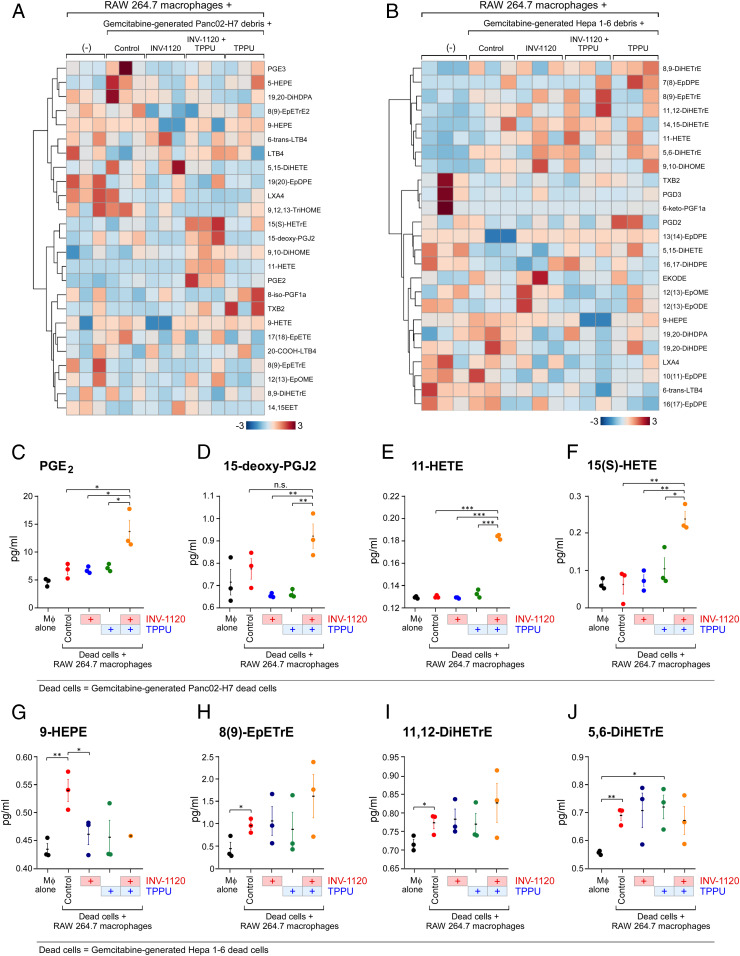
Combined inhibition of sEH and EP4 differentially regulates the release of eicosanoids by debris-stimulated macrophages. LC-MS/MS–based oxylipin analysis of conditioned medium from RAW 264.7 macrophages stimulated by gemcitabine-generated (*A*) Panc02-H7 or (*B*) Hepa 1-6 debris, macrophages treated with an sEH inhibitor (TPPU, 10 µM, 2 h) and/or EP4 antagonist (INV-1120, 10 µM, 2 h) and stimulated by gemcitabine-generated Panc02-H7 debris, gemcitabine-generated Hepa 1-6 debris, or macrophages not stimulated with debris (“macrophages [MØ] alone”). (*A* and *B*) Heat maps; (*C*–*J*) Quantification of 8 oxylipins in *A* and *B*, including PGE_2_, 15-deoxy-PGJ_2_, 11-HETE, 15(S)-HETE, 9-HETE, 8 (9)-EpETrE, 11,12-DiHETrE, and 5,6-DiETrE. Data are presented as means (pg/mL) ± SEM *n* = 3 per group. *P < 0.05, **P < 0.01, ***P < 0.001, n.s., not significant.

Inflammation stimulates the release of eicosanoids that, when uncontrolled, can lead to an “eicosanoid storm” that drives cytokine production (33, 35, 55, 103, 104). To determine whether chemotherapy-generated debris triggers the release of bioactive lipid autacoids by macrophages, we performed LC-MS/MS–based oxylipin profiling on the conditioned medium of RAW 264.7 macrophages stimulated by gemcitabine-generated Panc02-H7 debris (Fig. 4*A*) or Hepa 1-6 debris (Fig. 4*B*). Indeed, debris-stimulated macrophages released a storm of bioactive lipid mediators (eicosanoid storm) (Fig. 4 *A* and *B*), including 9-hydroxyeicosapentaenoic acid (9-HEPE), 8,9-epoxy-5,11,14-eicosatrienoic acid [8 (9)-EpETrE], 11,12-dihydroxy-5,8,14-eicosatrienoic acid (11,12-DiHETrE), and 5,6-DiHETrE, compared to macrophages not exposed to debris (Fig. 4 *G*–*J*). Treatment with an sEH inhibitor (TPPU) or an EP4 antagonist (INV-1120) stimulated lipid mediators that promote inflammation resolution, including PGE_2_, 15-deoxy-Δ^12,14^-prostaglandin J_2_ (15-deoxy-PGJ_2_), 11-hydroxyeicosatetraenoic acid (11-HETE), and 15(*S*)-HETE (Fig. 4 *C*–*F*). Notably, PGE_2_ can induce lipid mediator class switching from proinflammatory eicosanoids to proresolving lipid mediators (105). Therefore, combined inhibition of sEH and EP4 differentially regulates the release of lipid mediators.

### Combined Inhibition of sEH and EP4 Prevents Debris-Stimulated Liver Metastases.

**Fig. 5.**
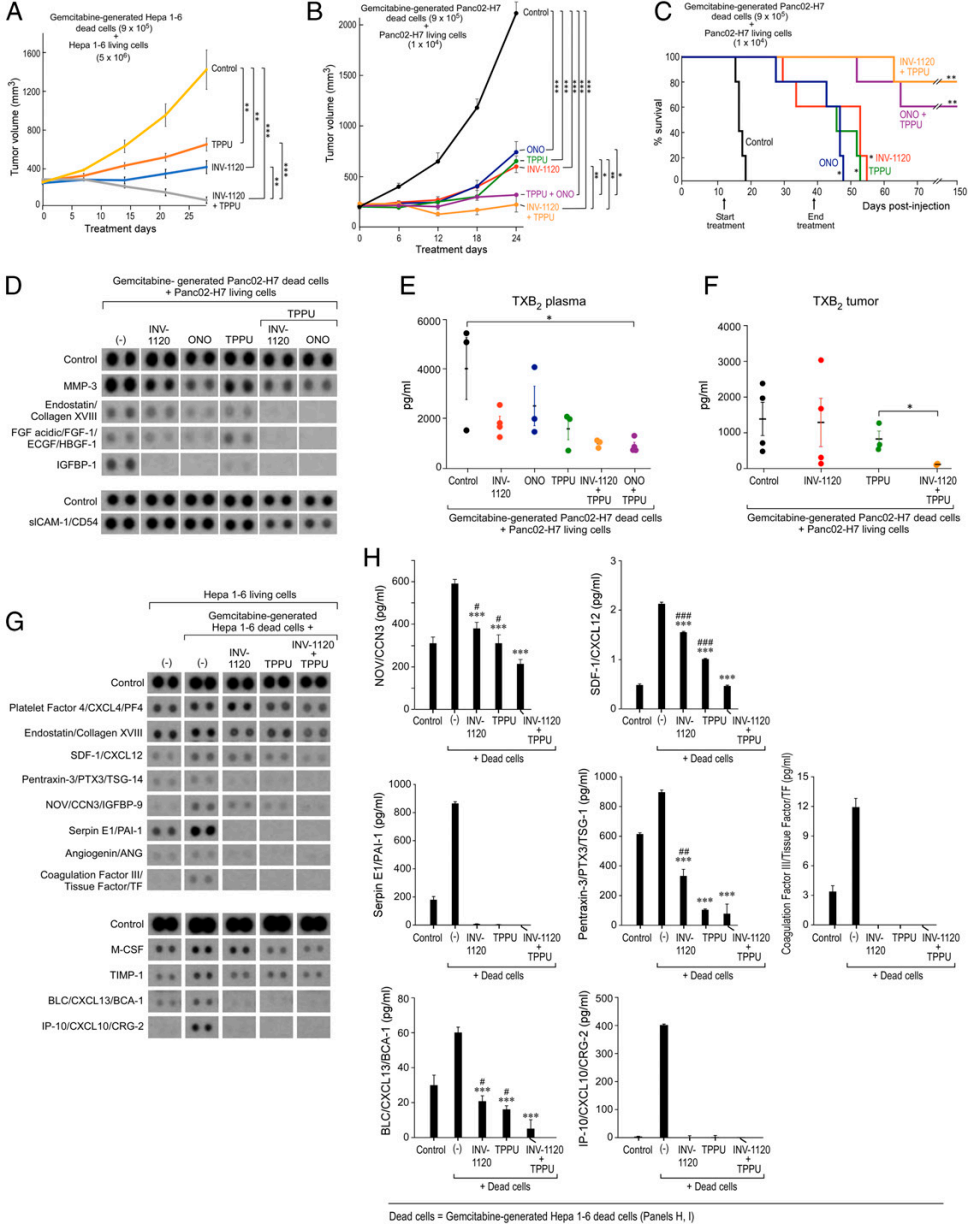
Prevention of debris-stimulated liver metastasis and cytokine storm by sEH inhibition and EP4 antagonism. (*A*) Growth of debris-stimulated tumors [gemcitabine-generated Hepa 1-6 debris [9 × 10^5^ dead cells] + Hepa 1-6 [5 × 10^6^ living cells]) systemically treated with TPPU (5 mg/kg/d), INV-1120 (5 mg/kg/d), or TPPU + INV-1120 (5 mg/kg/d each). Treatment initiated once tumors reached 100 to 200 mm^3^. *n* = 5 mice per group. **P < 0.01, ***P < 0.001. (*B*) Growth of debris-stimulated tumors (gemcitabine-generated Panc02-H7 debris [9 × 10^5^ dead cells] + Panc02-H7 [1 × 10^4^ living cells]) systemically treated with an sEH inhibitor (TPPU, 5 mg/kg/d), EP4 antagonist (INV-1120 or ONO-AE3-208, 5 mg/kg/d), or a combination of them (TPPU + INV-1120 or TPPU + ONO-AE3-208, 5 mg/kg/d each). *n* = 5 mice per group. *P < 0.05, **P < 0.01, ***P < 0.001. (*C*) Percent survival of mice coinjected orthotopically with gemcitabine-generated Panc02-H7 debris (9 × 10^5^ dead cells) and Panc02-H7 (1 × 10^4^ living cells). Mice were systemically treated with an sEH inhibitor (TPPU, 5 mg/kg/d), EP4 antagonist (INV-1120 or ONO-AE3-208, 5 mg/kg/d), or a combination (TPPU + INV-1120, TPPU + ONO-AE3-208, or TPPU + INV-1120, 5 mg/kg/d each). Kaplan–Meier analysis indicated significantly prolonged survival in treated mice compared to control. *n* = 5 mice per group. *P < 0.05, **P < 0.01 vs. control. (*D*) Angiogenic (*Upper*) and inflammatory (*Lower*) cytokines in plasma, as well as TXB_2_ in plasma (*E*) or tumor tissues (*F*), from mice bearing debris-stimulated subcutaneous Panc02-H7 tumors treated with TPPU, INV-1120, ONO-AE3-208, TPPU + INV-1120, or TPPU + ONO-AE3-208. Plasma and tumor tissues were collected on treatment day 24. TXB_2_ product was quantified by ELISA. Data are presented as means (pg/mL) ± SEM *n* = 3 per group. *P < 0.05. (*G*) Angiogenic (*Upper*) and inflammatory (*Lower*) cytokines of plasma from mice bearing debris-stimulated subcutaneous Hepa 1-6 tumors treated with TPPU, INV-1120, or TPPU + INV-1120. Plasma was collected on treatment day 28. (*H*) Quantification of cytokines by ELISA shown in *G*. Data were presented as means (pg/mL) ± SEM *n* = 3 per group. *P < 0.05, **P < 0.01, ***P < 0.001 vs. debris-stimulated tumors without treatment. ^#^*P* < 0.05, ^##^*P* < 0.01, ^###^*P* < 0.001 vs. INV-1120 + TPPU.

We next determined whether combined inhibition of sEH and EP4 could suppress chemotherapy-generated debris-stimulated hepato-pancreatic tumor growth. Systemic treatment with either TPPU, INV-1120, or ONO-AE3-208 suppressed debris-stimulated tumor growth (Fig. 5*A*). Notably, the combination of TPPU and INV-1120 or TPPU and ONO-AE3-208 induced tumor regression or stabilized growth of established debris-stimulated Hepa 1-6 or Panc02-H7 tumors without overt toxicity (Fig. 5 *A* and *B*). Importantly, TPPU and INV-1120 or TPPU and ONO-AE3-208 exhibited potent antitumor activity compared to monotherapy (e.g., TPPU, ONO-AE3-208, or INV-1120 alone) (Fig. 5 *A* and *B*). To exclude that the antitumor activity of combined sEH and EP4 inhibition was specific to subcutaneous tumors, we utilized an orthotopic Panc02-H7 debris-stimulated model in which the intrasplenic injection of gemcitabine-generated Panc02-H7 dead cells and Panc02-H7 living cells induced liver metastasis (Fig. 5*C*). Remarkably, systemic treatment with an sEH inhibitor (TPPU) and an EP4 antagonist (ONO-AE3-208 or INV-1120) prolonged survival compared to control or monotherapy (Fig. 5*C*). Compared to TPPU, INV-1120, or ONO-AE3-208 treatment alone, the combination treatment with an sEH inhibitor and an EP4 antagonist (TPPU and INV-1120 or TPPU and ONO-AE3-208) suppressed the proinflammatory and proangiogenic cytokine storm in vivo, including endostatin/collagen XVIII, acidic FGF/FGF-1/ECGF/HBGF-1, IGFBP-1, and soluble intercellular adhesion molecule-1 (slCAM-1)/CD54, in mice bearing debris-stimulated Panc02-H7 tumors (Fig. 5*D*). Additionally, the combination of TPPU and ONO-AE3-208 suppressed thromboxane B2 (TXB_2_) in plasma of mice bearing debris-stimulated Panc02-H7 tumors compared to control (Fig. 5*E*). TXB_2_ levels in tumor tissues were inhibited by combined treatment with TPPU and INV-1120 in mice bearing debris-stimulated Panc02-H7 tumors compared to treatment with TPPU alone (Fig. 5*F*). Moreover, the combination of TPPU and INV-1120 also inhibited the cytokine storm (e.g., SDF-1/CXCL12, PTX3/TSG-14, nephroblastoma overexpressed (NOV)/CCN3, IGFBP-9, angiogenin, BLC/CXCL13/BCA-1, interferon-gamma-induced protein (IP)10/CXCL10/cytokine responsive gene (CRG)-2 and Serpin E1/PAI-1) compared to control, TPPU treatment alone, or INV-1120 treatment alone in the plasma of mice bearing debris-stimulated Hepa 1-6 tumors (Fig. 5 *G* and *H*). Thus, combined treatment with an sEH inhibitor and an EP4 antagonist prevents debris-stimulated tumor growth, prolongs survival in a metastatic pancreatic cancer model, and counterregulates the debris-stimulated cytokine storm in vivo.

## Discussion

Most hepato-pancreatic cancer-related deaths are the result of metastatic recurrence (1, 2). Cancer therapy is a double-edged sword, as surgery, radiation, and chemotherapy can induce a proinflammatory injury response that promotes dormancy escape and tumor recurrence (42, 106). Targeting multiple arachidonic acid pathways may represent a new therapeutic approach to stimulating the active resolution of inflammation in cancer metastasis. Here, we identified a critical tumor-promoting and prometastatic role for chemotherapy-generated pancreatic and hepatocellular cancer cell debris, which triggers a storm of proinflammatory eicosanoid-driven cytokines via the upregulation of *Ephx2* and *Ptger4*. The combination of sEH and EP4 inhibition provides a unique antiinflammatory and debris-clearing approach by promoting phagocytosis of cellular debris by macrophages and counterregulating a series of proinflammatory and proangiogenic cytokines. Given the apparent nontoxicity of sEH inhibitors and EP4 antagonists (62, 65, 83), these compounds, which are currently in clinical development for multiple inflammatory diseases, could be rapidly translated to the clinic to be used in conjunction with surgery, chemotherapy, and radiation.

Cytotoxic cancer chemotherapy generates tumor cell debris, which disrupts the resolution of inflammation. Consequently, novel therapies are urgently needed to stimulate the clearance of protumorigenic tumor cell debris and counterregulate proinflammatory eicosanoids and cytokines. Moreover, preoperative inflammation blockade and stimulation of resolution eradicates micrometastases (42). Thus, administering inhibitors of sEH and EP4 before or during surgery, chemotherapy, and radiation may prevent cancer therapy-stimulated tumor growth and metastases. The combination of an sEH inhibitor with an EP4 antagonist exhibits synergistic antitumor and antimetastatic activity. Thus, simultaneously blocking the ensuing proinflammatory response and activating debris clearance programs with chemotherapy, surgery, and radiation may prevent tumor growth and metastases.

While intended to reduce tumor burden, chemotherapy, radiation, and surgery, can paradoxically stimulate tumor growth and subsequent metastatic outgrowth through inflammation and failure of inflammation resolution (8, 33, 55, 107–116). Consistent with studies based on the Révész effect, including the protumorigenic activity of apoptotic cell debris-mediated inflammation (8, 23–25, 27, 33–36, 39, 42, 49, 52, 53, 117–124), we demonstrate that dead and dying pancreatic and hepatocellular cancer cells killed by cancer chemotherapy (e.g., gemcitabine) can promote tumor growth and metastasis. Elevated spontaneous apoptotic cell death rates in cancer patients’ tumors, including liver (e.g., HCC), ovarian, head and neck (e.g., laryngeal and esophageal), breast, prostate, synovial sarcoma, non-Hodgkin’s lymphoma, carcinoma of the tongue and lymph node metastasis, and bladder correlate with a poor prognosis [as reviewed in Ucker and Levine (125)] (41, 43–46, 51, 126–131). As tumors increase in size, cell death is more frequent (132). Biomarkers of tumor cell debris (e.g., caspases) may predict tumor recurrence and metastases (125). Interestingly, tumor cell debris promotes pancreatic cancer metastasis by inducing inflammation through TAMs (36). Thus, the failure to clear apoptotic and necrotic cell debris within tissues can perpetuate the inflammatory response, resulting in poor clinical outcomes.

To prevent tumor recurrence after chemotherapy, it is critical to effectively target the inflammatory response and the tumor-promoting activity of therapy-generated debris. The side effects of antiinflammatory drugs, including steroids and nonsteroidal antiinflammatory drugs, such as immunosuppression, impaired wound healing, bleeding, and cardiovascular toxicity, prevent their widespread chronic use (106). Stimulation of inflammation resolution via resolvins and neutrophil-dependent monocytes and macrophage polarization inhibit tumor growth (8, 117, 133, 134). Arachidonic acid metabolized by cytochrome P450 (CYP450) enzymes produces EETs that exhibit antihypertensive, antiinflammatory, analgesic, and cardioprotective activity (135) and also stimulate inflammation resolution by promoting the macrophage-driven clearance of cell debris (59, 60). In recent years, a new direction has emerged in inflammation research with the discovery of sEH inhibitors, which stabilize EETs, counterregulate proinflammatory cytokines, and exhibit potent debris-clearing activity (33, 35, 62, 65). Notably, bioactive SPMs also play a critical role in preventing debris-stimulated tumor growth (8, 106, 136), and CYP450-derived EETs stimulate the production of SPMs, such as lipoxin A_4_ (63, 137). Importantly, sEH inhibition reduces inflammation in mouse models of multiple diseases, including atherosclerosis, abdominal aortic aneurysm, dyslipidemia, hypertension, and diabetes (62, 65).

**Fig. 6.**
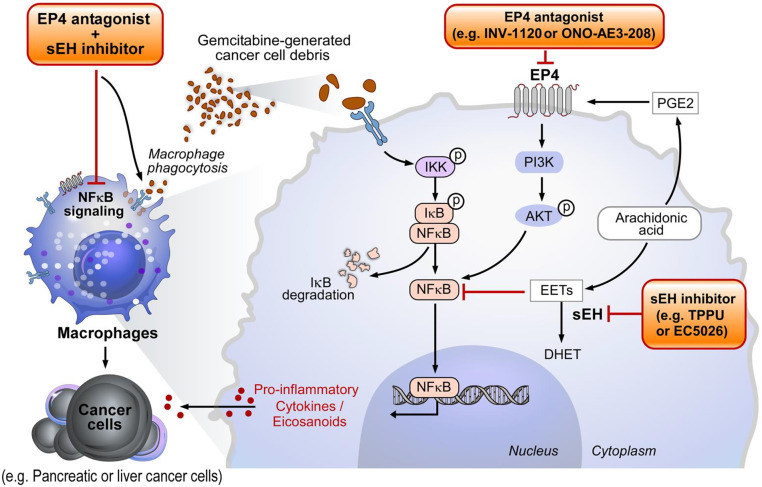
Potential mechanism for the resolution of debris-stimulated metastatic hepato-pancreatic cancer via combined soluble epoxide hydrolase and EP4 inhibition.

Likewise, the antiinflammatory and proresolution properties of EP4 antagonists have been harnessed to enhance the antitumor activity of chemotherapy by inducing the clearance of cancer cells by extracellular vesicles (95). While macrophages and neutrophils are key players in resolving inflammation as effector cells that kill microbes and clear debris (55), PGE_2_ and EP4 activation impairs immune host defenses by suppressing phagocytosis. PGE_2_ inhibits antitumor immunity via suppression of antigen presentation, thereby inhibiting NK and T cell function (138–140). This study demonstrates that combined pharmacological abrogation of the sEH and EP4 pathways inhibits chemotherapy-induced cancer progression by preventing the protumorigenic cytokine storm via clearance of tumor cell debris (Fig. 6).

Although the generation of tumor cell debris and inflammation may be an inherent limitation of current cancer therapy, overcoming the dilemma of debris-induced tumor progression with the inhibition of sEH and EP4 represents a promising approach to prevent hepato-pancreatic tumor growth, metastasis, and recurrence. While the antitumor activity of sEH and EP4 inhibition has yet to be evaluated in humans, this approach to inflammation resolution may allow for the restoration and maintenance of proresolving processes in between cycles of cytotoxic cancer therapies. Thus, synergistic antimetastatic activity via inhibition of sEH and EP4 may be a host-directed therapeutic approach to enhance the endogenous clearance of tumor cell debris and address the intrinsic limitation of cytotoxic cancer therapy.

## Materials and Methods

### Cell Culture and Drugs.

RAW 264.7 murine macrophages (ATCC) and Panc02-H7 murine pancreatic ductal adenocarcinoma cells (ATCC) were cultured in Dulbecco’s modified Eagle medium (ATCC). Hepa 1-6 murine HCC cells (ATCC) were cultured in Roswell Park Memorial Institute 1640 (RPMI) medium (ATCC). HepG2 human hepatocellular carcinoma cells (ATCC) were cultured in Eagle’s minimum essential medium (ATCC). hMDMs were isolated from blood provided by healthy volunteers at the Children’s Hospital Boston blood bank using density-gradient Histopaque-1077 (Sigma-Aldrich). Human monocytes were differentiated into macrophages using RPMI supplemented with 10 ng/mL GM-CSF (R&D Systems) for 7 d as described previously (35). All cell culture media were supplemented with 10% fetal bovine serum (FBS; Thermo Fisher Scientific) and 1% l-glutamine-penicillin-streptomycin (GPS; Thermo Fisher Scientific). Gemcitabine was purchased from Sigma-Aldrich. ONO-AE3-208 (EP4 antagonist) was purchased from Cayman Chemical.

### Generation of Tumor Cell Debris.

Tumor cell debris was generated by refeeding 70% confluent T150 flasks with complete medium containing 10% FBS and 40 μM gemcitabine (Sigma-Aldrich) and incubating for 72 to 96 h at 37 °C, as described previously (35).

### Animal Studies.

All animal studies approved by the Animal Care and Use Committee of Beth Israel Deaconess Medical Center, Boston, MA. Six-week-old C57BL/6 male mice (The Jackson Laboratory) were housed under a 12-h day light cycle at a maximum of five mice per cage in a pathogen-free facility with free access to sterile water and chow. Mice were injected with debris and/or living tumor cells, as described previously (35). For subcutaneous injections, gemcitabine-generated Panc02-H7 or Hepa 1-6 tumor cell debris (9 × 10^5^ dead cells) was combined with Panc02-H7 (1 × 10^4^ living cells) or Hepa 1-6 (5 × 10^6^ living cells) at equal volumes in PBS (100 µL per mouse), respectively. For the orthotopic liver metastasis model, mice were injected directly into the spleen with Matrigel (20 µL per mouse) (Becton Dickinson and Company) mixed with gemcitabine-generated Panc02-H7 debris (9 × 10^5^ dead cells) and Panc02-H7 (1 × 10^4^ living cells) at a 1:1 (Matrigel:cells) ratio. Treatment of mice with TPPU (5 mg/kg/d), INV-1120 (5 mg/kg/d), ONO-AE3-208 (5 mg/kg/d), TPPU + ONO-AE3-208 (5 mg/kg/d each), TPPU + INV-1120 (5 mg/kg/d each), or control (DMSO + PEG400) was performed by miniosmotic pumps (Alzet Inc.) implanted into the peritoneum of mice on the day of tumor cell injection.

### Quantitative Real-Time PCR.

Tumor tissue RNA was isolated according to the protocol provided in the Qiagen Multiplex PCR Plus Kit (Qiagen). The total RNA was then reverse-transcribed into cDNA using the High-Capacity cDNA Reverse Transcription kit (Applied Biosystems). Quantitative real-time PCR (qRT-PCR) was performed by CFX qPCR instruments (Bio-Rad) with Maxima SYBR-Green Master Mix (Thermo Fisher Scientific). The expression levels of target genes were quantified by normalization of GAPDH gene using the 2^−ΔΔCt^ method, as described previously (35). The primers of genes are listed in *SI Appendix*, Table S1.

### Immunoblotting.

Macrophages (RAW 264.7 or hMDMs) were treated with INV-1120 (10 µM), TPPU (10 µM), or a combination of both for 2 h, and then coincubated with gemcitabine-generated Hepa 1-6 debris in complete medium for 1 h. Macrophages were then incubated in fresh serum-free medium overnight at 37 °C. The medium was decanted, and the cells were washed with cold PBS and lysed with RIPA buffer plus phosphatase inhibitor (Sigma-Aldrich). Hepa 1-6 liver tumor tissues from the in vivo study were collected and incubated at −80 °C. 30 mg of tumor tissue was lysed with RIPA buffer plus phosphatase inhibitor (Sigma-Aldrich). Protein concentration was determined by the Pierce BCA protein assay kit (Thermo Scientific). The membranes were blocked in 5% blotting-grade blocker (Bio-Rad, cat #1706404) for 1 h at room temperature and incubated with primary antibodies overnight at 4 °C. The membranes were then incubated with secondary antibodies for 1 h and then imaged by SuperSignal West Pico Plus Luminol/Enhancer (Thermo Scientific, cat #34577) and filmed in a photo processor, as described previously (35). All antibodies used in this study were from Cell Signaling Technology, including IKKβ (1:1,000; cat #8943), phospho-IKKβ (Ser176/180) (1:500; cat #2697), NF-κB (1:1,000; cat #8242), phospho–NF-κB (Ser536) (1:1,000; cat #3033), AKT (1:1,000; cat #4691), phospho-AKT (Ser473) (1:1,000; cat #4060), β-actin (1:5,000; cat #3700), HRP-linked anti-mouse IgG (1:3,000; cat #7076), and HRP-linked anti-rabbit IgG (1:3,000; cat #7076). The protein expression levels were quantified by Image J (NIH).

### Cytokine Array and ELISA Assay.

Macrophages (RAW 264.7 or hMDMs, 1 × 10^6^ cells per well) were plated in six-well plates and cultured in PBS with calcium and magnesium (PBS^+/+^) for 2 h at 37 °C, as described previously (35). Macrophages were treated with vehicle (DMSO), EP4 antagonist (INV-1120 or ONO-AE3-208), sEH inhibitor (TPPU or EC5026), or a combination of both at different concentrations (0 to 20 μM) for 2 h at 37 °C. Tumor cell debris was generated as described previously (35). Macrophages were incubated with debris at a 1:4 macrophage-to-debris ratio for 1 h. Macrophages were then incubated in fresh serum-free medium overnight at 37 °C. Conditioned media was centrifuged (1,100 rpm, 5 min) to remove particulates and stored at −80 °C or analyzed immediately. Media or mouse plasma was analyzed via Proteome Profiler kits or ELISA kits (R&D Systems): mouse cytokine/angiogenesis array, human cytokine/angiogenesis array, according to the kit protocol. Array control allowed for comparison between membranes. The TXB_2_ product from mouse plasma or tumor tissues was analyzed by a TXB_2_ ELISA kit (Cayman Chemical).

### Macrophage Phagocytosis Assay.

Macrophages (RAW 264.7 or hMDMs, 5 × 10^4^ cells per well) were plated in 96-well plates and cultured in complete RPMI medium for 18 to 24 h, followed by PBS for 1 to 2 h at 37 °C before treatment with drugs, as described previously (35). Tumor cell debris was prepared as described previously (35) and fluorescently stained with carboxyfluorescein diacetate (CFDA). Macrophages were treated with vehicle (DMSO), EP4 antagonist (INV-1120 or ONO-AE3-208, 10 μM), sEH inhibitor (EC5026 or TPPU, 10 μM), or a combination of both for 2 h at 37 °C. CFDA-stained debris (HepG2 dead cells for hMDM phagocytosis assays or Hepa 1-6 dead cells for RAW 264.7 murine phagocytosis assays) was added to 96-well plates at a 1:4 macrophage to debris ratio. After incubation for 1 h at 37 °C, plates were quenched with Trypan blue, and fluorescence was measured by a Spectra Max M5 plate reader (Molecular Devices). Relative fluorescence units (RFU) were used to measure phagocytosis.

### Oxylipins Analysis.

Conditioned medium from each well was collected and centrifuged (1,100 rpm for 5 min) to remove particulates and stored at −80 °C or analyzed immediately, as described previously (35). Oxylipins of cell media were extracted according to a previous protocol (66). The oxylipins were analyzed and quantified by LC-MS/MS analysis.

### Statistics.

All data were presented as mean ± SEM. Statistical significance of differences were analyzed by Student’s *t* test between two groups and one-way ANOVA among more than two groups. The Kaplan–Meier product-limit method was used to evaluate survival differences over time after the day of tumor cell injection between mice coinjected with tumor cell debris and living cells vs. living cells alone. *P* values less than 0.05 considered statistically significant.

## Supplementary Material

Supplementary File

## Data Availability

All study data are included in the main text and *SI Appendix*.
