# Supplementary material for: Eicosanoid regulation of debris-stimulated metastasis
Source: Proc Natl Acad Sci U S A. 2021 Oct 4;118(41):e2107771118. doi: 10.1073/pnas.2107771118 (PMC8521662; doi:10.1073/pnas.2107771118)
Supplement: Supplementary File [file pnas.2107771118.sapp.pdf]

Supplemental Figure 1

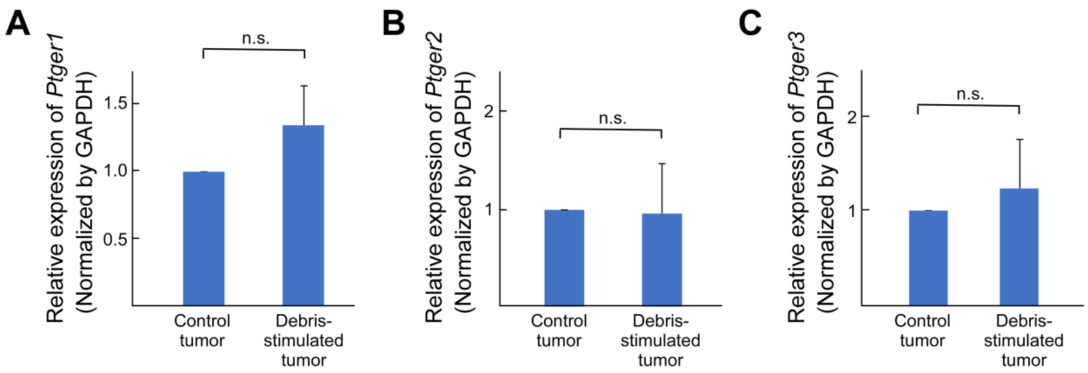

Supplemental Figure 2

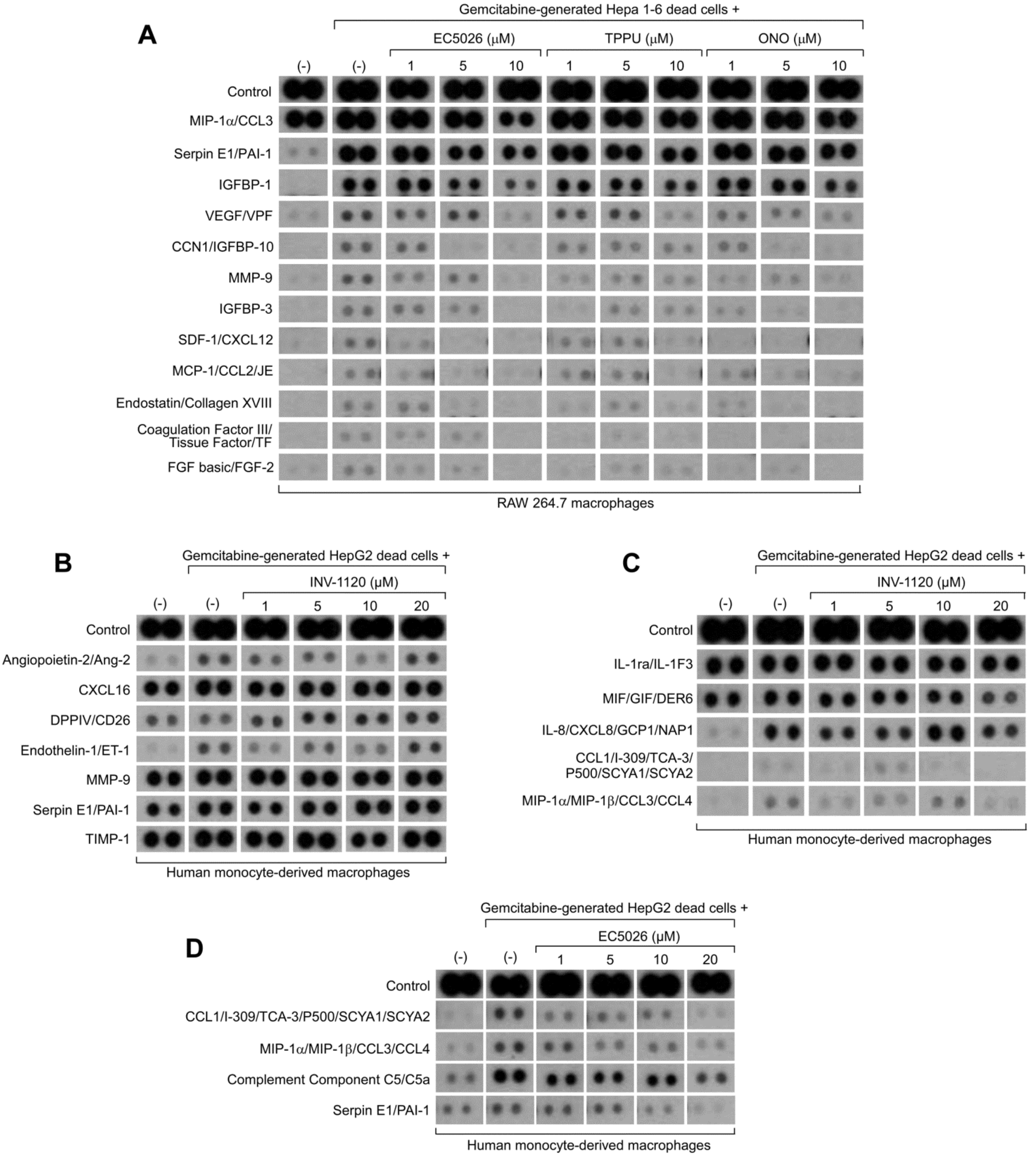

Supplemental Figure 3

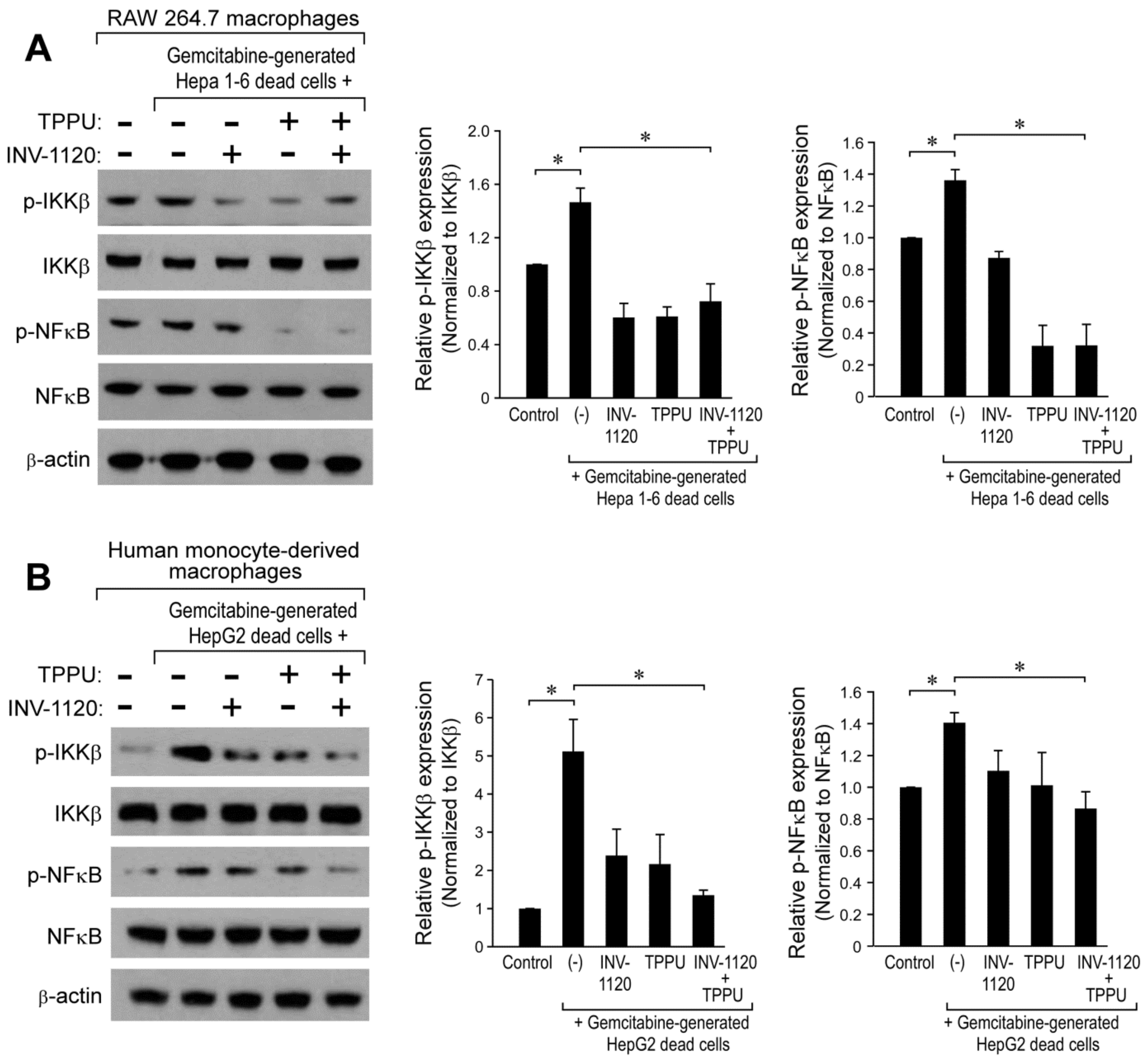

Supplemental Table 1

Supplementary Table S1. Primer sequences of target genes.

Name	Sequence (5'-3')	Reference
Ptger1	CTCCTTGCGGCATTAGTGTG (F)	[1]
	TGCGGTCTTTCGGAATCGT (R)	[1]
Ptger2	CGTTATCCTCAACCTCATTCGC (F)	[1]
	TCCGTCTCCTCTGCCATGG (R)	[1]
Ptger3	TTGCTGGCTCTGGTGGTGAC (F)	[1]
	GCTGGACTGCGAGACGGC (R)	[1]
Ptger4	TGACCCAAGCAGACACCACCT (F)	[1]
	TCCCACTAACCTCATCCACCAA (R)	[1]
Ephx2	CTGTGGCCAGTTTGAACACG (F)	[2]
	ATCACTGGCTCGGAAGAAGC (R)	[2]
**GAPDH**	AGGTCGGTGTGAACGGATTTG	[3]
	TGTAGACCATGTAGTTGAGGTCA	[3]

F: forward primer; R: reverse primer.

[1] Dejani *et al.*, Intestinal host defense outcome is dictated by PGE2 production during efferocytosis of infected cells. *Proc Natl Acad Sci* U S A 115, E8469-E8478 (2018).

[2] Yang *et al.*, Estrogen-dependent epigenetic regulation of soluble epoxide hydrolase via DNA methylation. *Proc Natl Acad Sci* U S A 115, 613-618 (2018).

[3] Yang *et al.*, A common antimicrobial additive increases colonic inflammation and colitis-associated colon tumorigenesis in mice. *Sci. Tranl Med* 443, eaan4116 (2018).

Supplemental Table 2

A

Sample/Treatment Name	RAW 264.7 macrophages			Dead cells			RAW 264.7 macrophages + Dead cells + INV-1120			RAW 264.7 macrophages + Dead cells +TPPU			RAW 264.7 macrophages + Dead cells + INV-1120 + TPPU			RAW 264.7 macrophages + Dead cells + EC5026		
Group	1	2	3	1	2	3	1	2	3	1	2	3	1	2	3	1	2	3
12(13)-EpODE	0.06572127	0.1628483	0.13762306	0.0810586	0.04946725	0.01989588	0.02993439	0.04799161	0.08233717	0.08497168	0.01283646	0.12048517	0.28822463	0.02149612	0.0106862	0.01192464	0.03053199	0.12011641
13-HOTrE	n.d.	2.1745811	0.92103113	1.45037119	1.45918299	n.d.	0.37978785	7.16610591	1.67419661	0.96090085	1.34318172	4.9203523	0.77552328	1.47240173	1.49357907	n.d.	n.d.	1.72016164
12(13)-EpOME	6.40826315	4.52542431	10.2514882	4.99736496	3.26048533	5.11998767	4.01147313	4.55906219	3.61888723	3.44785011	2.58066926	5.14270417	7.78001987	4.91993915	9.04658542	5.80646843	4.09365786	12.5604237
9(10)-EpOME	4.57312337	4.12632061	4.550976	5.37153094	3.65234349	4.05023223	3.37884777	4.00601019	3.61201934	2.95103102	3.20669706	5.55696881	4.59172197	5.22889609	5.81591243	4.48571299	3.35614372	11.3551926
EKODE	3.44777217	2.94328552	3.99627664	3.73182266	3.28755238	1.66050967	2.44665753	1.66942053	1.66876132	6.91587864	0.34029575	4.78337408	7.59370346	5.87374888	2.67328947	2.39017037	5.54767872	5.74826463
12,13-DiHOME	0.3227096	0.4041285	0.39419846	0.56720009	0.19173022	0.26681905	0.30455372	0.23390875	0.36933815	0.39007289	0.32604894	0.3758358	0.38522677	0.51513514	0.23519894	0.33784584	0.20399419	0.4982936
9,10-DiHOME	0.04272244	0.09537784	0.15476141	0.11001743	0.0864127	0.16769737	0.15565181	0.13756014	0.13527787	0.17474792	0.15313212	0.17644439	0.17578106	0.22876796	0.18119242	0.23839403	0.11372709	0.28957644
15-deoxy-PGJ2	0.63244776	0.68673537	0.82605076	0.68882424	0.78818546	0.84713731	0.66561979	0.64754416	0.65177631	0.65630725	0.65127992	0.68374428	0.83622369	0.90497603	1.02284467	0.98551021	0.66603927	0.64542545
14(15)-EpETE	n.d.	0.2938228	0.24018117	0.25402546	0.27068914	0.24034076	0.25575433	0.25516253	0.25675176	0.24080623	0.26701196	0.23990189	0.25377278	0.2561533	0.25669191	0.25566124	0.27078224	0.24272129
11(12)-EpETE	0.11797235	0.08816573	0.107438	0.08835109	0.05366351	0.12175677	0.06835089	0.08942239	0.08954506	0.0678898	0.08956191	0.08882283	0.10081854	0.14211168	0.08804511	0.07164373	0.10588921	0.05109371
17(18)-EpETE	0.51939595	0.45944234	0.59230759	0.64795286	0.77480955	0.53667883	0.57022116	0.42205387	0.65491884	0.40483702	0.5696104	0.4216577	0.40176671	0.56525254	0.70828613	0.70726269	0.62758316	0.79750674
5-HEPE	0.46720202	0.46936791	0.46405086	0.49456233	0.48429876	0.47350328	0.47423055	0.46600229	0.46496924	0.47381755	0.47207315	0.48704966	0.47622657	0.46814588	0.4654502	0.4609772	0.47225683	0.47345126
8-HEPE	0.59371442	0.64660547	0.58795997	0.61263658	0.53089988	0.64609168	0.58794529	0.61234299	0.58377625	0.61234299	0.58452491	0.55827756	0.62388125	0.6769191	0.55742614	0.61755429	0.67649339	0.55516546
9-HEPE	0.454967	0.42896284	0.47845383	0.42780368	0.4289768	0.42674229	0.45217386	n.d.	n.d.	0.42582055	0.45440838	0.48246972	0.42777575	0.45813345	n.d.	0.45197491	0.4785096	n.d.
11-HETE	0.12965465	0.12827243	0.12871403	0.12910392	0.1306688	0.12948357	0.12889586	0.12820485	0.12923343	0.12947996	0.13182041	0.13649867	0.18074727	0.18532749	0.18409296	0.19601729	0.20651161	0.19086161
14,15-EET	0.69801504	0.83049292	0.5740423	0.27124464	0.21078915	0.46408322	0.4627192	0.30267961	1.04687254	0.35426918	0.37402955	0.38054354	0.67781776	0.53517883	0.35104061	0.63005532	0.59336496	1.70448189
15-HETE	0.30054695	n.d.	0.45502939	0.61385747	0.43236658	0.31063711	0.3948163	0.21348767	0.22006068	0.25499256	0.26819987	0.5453536	0.20909996	0.17627362	0.27745823	0.1575312	0.41054663	0.24766774
5-HETE	0.22349249	0.22234679	0.22350976	0.23165849	0.24912144	n.d.	n.d.	0.2212126	0.22121317	0.22232548	0.22116424	0.25267956	0.23047835	0.22465374	0.22698827	0.22699116	0.22577123	0.22935228
8(9)-EpETrE 2	6.38812223	9.16593203	7.30289283	5.47486322	6.57345043	10.8309166	0.74170283	4.50329301	1.12051066	5.50733844	8.87688442	4.10651176	n.d.	6.55937067	6.49739466	7.44871197	12.3267029	5.59329397
8(9)-EpETrE	1.65340298	0.31745125	2.17573478	0.41216325	0.17275309	0.42273222	0.74610523	0.21563131	0.18318893	0.1626061	0.14627356	0.7951555	0.20542953	0.33644029	0.93928055	2.07057673	0.82028363	1.39354416
9-HETE	0.08382843	n.d.	0.09080786	0.08714602	0.09819315	0.08349142	n.d.	n.d.	0.08727828	0.09538036	0.08323051	0.09098724	0.09075169	0.08348961	0.08323051	0.10194002	0.0871587	0.0906013
15(S)-HETrE	0.05886296	0.05185035	0.07890663	0.00965586	0.08688866	0.09096977	0.04622564	0.10039208	0.07195599	0.07954395	0.07238499	0.16270221	0.2211751	0.21597154	0.27940214	0.10342425	0.07572508	0.03173518
9,10,13-TriHOME	n.d.	n.d.	n.d.	0.25813805	n.d.	n.d.	n.d.	n.d.	n.d.	n.d.	n.d.	n.d.	n.d.	n.d.	n.d.	0.14246931	n.d.	n.d.
9,12,13-TriHOME	0.47933045	n.d.	0.7979735	0.64237626	0.72137871	0.31978557	n.d.	0.29397875	n.d.	n.d.	0.25974958	0.32276167	n.d.	n.d.	n.d.	n.d.	0.07918379	n.d.
11,12-DiHETE	0.60266312	0.60573444	0.60766439	0.62692687	0.62088094	0.60842185	0.61526747	0.61306255	0.59339467	0.61015206	0.59945172	0.61343091	0.60506778	0.6156488	0.6327045	0.60341538	0.60575	0.61825399
14,15-DiHETE	0.64281027	0.64834146	0.64004565	0.65383717	0.64798404	0.62977222	0.62729274	0.6380387	0.62757238	0.6273453	0.64337657	0.62731568	0.62749402	0.67040974	0.65324123	0.63811192	0.67432324	0.6417953
17,18-DiHETE	0.57753317	0.60363476	0.58997985	0.62965473	0.57438958	0.60093086	0.56001013	0.62390721	0.55837181	0.57592259	0.57850326	0.60614066	0.58506029	0.58453381	0.58968681	0.57586661	0.57720066	0.57690856
5,15-DiHETE	0.66655306	0.68536911	0.65493293	0.79939332	0.67200902	0.70368678	0.65494167	0.73962264	0.8609596	0.66283707	0.65356894	0.65331538	0.68304334	0.68945233	0.6375683	0.68812332	0.65488047	0.6349365
6-trans-LTB4	5.99507556	5.40963139	6.54234703	5.51239832	4.97613614	5.50024901	6.12554815	6.99338568	4.7418965	5.55192547	6.01696916	5.56879124	5.71099484	4.92487129	6.13378869	4.98600377	4.79383437	4.07947343
LTB4	11.7866236	8.59958144	9.66656463	7.14158426	6.4274439	8.6836077	8.82304298	9.96310345	7.99131677	10.4341172	9.19438082	6.41389279	9.25936117	9.40509422	10.6530665	7.20721646	8.64447136	8.01794153
11,12-DiHETrE	0.7156571	0.73528756	0.65302375	0.7242006	0.69753902	0.67441172	0.70070859	0.70553906	0.71573497	0.68497274	0.69038753	0.67020959	0.67195418	0.67698677	0.72360712	0.71416102	0.6627985	0.67047553
14,15-DiHETrE	0.38597199	0.46302145	0.39808037	0.44652517	0.35958633	0.36897533	0.45548555	0.36811396	0.37704289	0.37202012	0.36779428	0.41414006	0.37962889	0.37582176	0.36718709	0.36706214	0.37987004	0.37765778
5,6-DiHETrE	0.55940302	0.60056177	0.59282415	0.63292367	0.65141659	0.54209101	0.57354056	0.56675399	0.58875177	0.58801287	0.57242548	0.58992036	0.57638285	0.71123185	0.62020775	0.56144937	0.61879698	0.64749631
8,9-DiHETrE	0.69636863	0.86338863	0.71767471	0.84972079	0.70169282	0.78669461	0.71705238	0.68494529	0.72526445	0.77821649	0.71118109	0.77184723	0.77620168	0.70612108	0.63227825	0.9872035	0.73139838	0.9168593
10(11)-EpDPE	0.45476852	0.49395671	0.43758943	0.48051166	0.45530662	0.49221983	0.46409479	0.48102379	0.54053756	0.476741	0.46207276	0.46322788	0.46632004	0.63999753	0.44394647	0.44750255	0.47303396	0.49603831
13(14)-EpDPE	0.87377848	0.85949403	0.85995925	0.86141433	0.85254052	0.91514195	0.86441741	0.86074565	0.85383469	0.91585559	0.84276146	0.87034	0.89931734	0.87031351	0.8559995	0.87072622	0.85011092	0.88114111
16(17)-EpDPE	0.70102611	0.70775258	0.69969366	0.72862633	0.67627942	0.71376066	n.d.	0.70394386	0.69708895	0.70568969	0.6949779	0.67743528	0.71819949	0.72898353	0.73983579	0.7158677	0.69498191	0.71577539
16(17)-EpDPE 2	0.76436748	0.7699298	0.79545742	0.79144618	0.79716192	0.78926443	0.81458467	0.78577022	0.80916155	0.76987015	0.75854947	0.81298813	0.76892131	0.76929346	0.7955256	0.8137239	0.7679668	0.76993833
19(20)-EpDPE	1.03484023	0.7182982	1.02942188	0.32142829	0.44595812	0.62992226	0.23163021	0.3557526	0.64606755	0.39372404	0.41190656	0.28097886	0.29098636	0.1571269	0.84497545	0.50558545	0.38213748	0.39546859
7(8)-EpDPE	21.4193801	n.d.	n.d.	n.d.	n.d.	n.d.	n.d.	n.d.	n.d.	13.1417207	n.d.	n.d.	n.d.	15.9008487	n.d.	23.1803453	1.68624716	n.d.
PGD3	2.33114272	2.17597953	2.80073009	2.80068005	4.28353497	2.60285545	2.44244891	2.30686543	2.21194159	2.23794823	2.3185427	3.34438349	3.33701924	2.87611031	3.21993424	2.67725183	3.86746098	2.22513282
PGE3	2.88838613	2.71825712	2.67208479	3.40169654	5.04948061	2.80708464	2.48314287	2.41416406	2.56383501	2.46957737	2.91544068	3.40157439	2.69939842	2.99611785	2.53736746	2.8071454	3.22554229	2.64399267
Resolvin	1.11822683	1.0867616	1.25384408	1.55178415	0.87880949	1.07956149	1.04003986	1.30820855	0.94961075	1.23746582	1.03481577	0.76213998	1.20121218	0.60026707	0.9736487	0.84239001	1.0643483	1.14049556
LXA4	2.28418761	1.97072728	2.55290973	2.40910286	1.4019354	1.33545917	1.24853245	1.36723244	1.86180324	1.12717393	1.15497821	1.62832144	1.21072214	1.23534232	1.0721003	1.61923273	1.03655807	1.48651652
PGD2	5.02754577	4.92742502	4.89385493	5.07408377	6.914279	14.729782	4.3864599	5.70679811	13.9085691	5.20450521	10.7400292	10.1755963	6.30109779	14.5899246	12.7977593	11.1931455	13.6609804	8.1172149
PGE2	4.88094347	5.14448019	3.8183885	5.2680454	6.90079697	7.87995076	6.27058665	6.62114989	7.42130895	7.10964693	6.59233288	7.87090893	17.7505898	12.0180222	11.3857669	13.8296109	12.7415125	12.8269374
8-iso-PGF1a	162.494208	17.3448116	204.825848	36.200404	54.9480427	n.d.	88.4650579	170.355029	92.6041042	123.350793	233.511369	328.693948	38.0460421	53.9925738	34.189145	242.668469	79.5668843	205.679614
PGF2a	1.38261214	1.67735819	1.08986442	2.18942822	1.2569746	2.98133494	2.247183	1.62045381	1.71187547	2.37941554	0.98053255	1.47369892	6.31573246	2.51029453	0.83262286	0.99102523	1.45877006	1.57129688
19,20-DiHDPA	0.50933578	0.45931697	0.41774287	0.67151084	0.48944511	0.45262248	0.46080675	0.44451799	0.35832968	0.37143049	0.35360579	0.46753581	0.44493718	0.43891158	0.37856206	0.41746937	0.33553867	0.37655958
10,11-DiHDPE	0.67233041	0.65584166	0.65289706	0.64092316	0.66261238	0.66323821	0.6632735	0.64777193	0.65399009	0.65504014	0.66954009	0.65573911	0.66088175	0.64598855	0.66267764	0.64262601	0.64800698	0.6627722
13,14-DiHDPE	0.53604697	0.53380718	0.54150148	0.54636033	0.54027443	0.53166298	0.53988467	0.53830463	0.5315096	0.5316031	0.54176622	0.53396266	0.53573916	0.53790226	0.53182687	0.53575597	0.53621821	0.53364119
16,17-DiHDPE	0.49222633	0.54691706	0.49903149	0.49996992	0.49150878	0.49872008	0.49873841	0.49164496	0.51700603	0.48448698	0.49204798	0.48449802	0.50010388	0.50372273	0.48166807	0.48905909	0.49962011	0.48091503
19,20-DiHDPE	0.38252611	0.42311364	0.38560882	0.43670381	0.40071295	0.42898616	0.43905607	0.41184852	0.38595984	0.54546596	0.41179492	0.34952669	0.39603041	0.42238468	0.47192726	0.39743164	0.43376128	0.45353618
4,5-DiHDPE	1.46374195	n.d.	3.51254657	2.27006932	n.d.	0.28859978	1.11586596	0.98950728	3.70497014	2.98447942	2.68399239	3.20787178	1.30675959	0.4917756	2.08653364	3.11361334	n.d.	0.49067618
7,8-DiHDPE	0.32995129	0.31901782	1.86383931	0.88390874	0.34219039	0.72179326	0.91875855	0.54640344	0.84005324	0.32327945	1.07877336	0.73022504	0.56172417	0.85565037	0.9803651	1.52185228	1.15276274	1.28669825
20-COOH-LTB4	2.28804433	1.77599154	3.1560239	2.54378642	2.04148699	1.61266214	2.95632876	1.89639742	2.49678944	2.40644512	3.21381524	1.75520242	1.90487428	2.19254432	2.19659543	4.10824298	2.77245724	2.73861118
TXB2	0.92581345	0.75545517	2.04426794	0.8024193	0.79681843	0.6321629	0.59055924	0.97720721	0.98737463	3.89476547	0.51359453	3.72085542	1.56384371	2.45147312	1.14251871	2.06695137	0.66976278	1.38939676
6-keto-PGF1a	1.33613117	1.20281597	1.24772943	1.21796142	2.85746589	1.29598348	1.27525786	1.48441593	1.25178076	1.27053091	1.23593442	2.60212553	1.56508861	1.43321955	1.34044072	1.32777938	1.22254457	1.38676589

Dead cells = Gemcitabine-generated Panc02-H7 dead cells

B

Sample/Treatment Name	RAW 264.7 macrophages			Dead cells			RAW 264.7 macrophages + Dead cells + INV-1120			RAW 264.7 macrophages + Dead cells +TPPU			RAW 264.7 macrophages + Dead cells + INV-1120 + TPPU		
Group	1	2	3	1	2	3	1	2	3	1	2	3	1	2	3
12(13)-EpODE	0.14406829	0.12866236	0.02914979	0.01262572	0.03957781	0.0392992	0.21589791	0.07204161	0.02986247	0.01312918	0.14486501	0.07364074	0.01933257	0.03876738	0.00399582
13-HOTrE	n.d.	1.0695273	2.25342689	0.08510999	5.05835859	1.14183905	n.d.	1.30454998	1.73041136	0.96537664	n.d.	1.42880693	n.d.	0.187431	n.d.
12(13)-EpOME	5.31616071	7.47681426	8.56771807	6.31433808	7.24502166	6.25149343	10.3024651	7.37605269	7.52909083	5.52287724	8.6138113	6.73186891	4.97579877	5.05199573	6.07543141
9(10)-EpOME	5.24995979	7.35134468	5.5453377	4.29199926	5.22080435	5.48864553	5.53698998	5.51540583	5.86117191	4.34908591	6.47166321	4.79713587	5.06109859	5.45583672	6.27175609
EKODE	2.83759622	0.96870237	1.22110089	5.04095299	2.56101183	2.0324243	4.59266506	8.98207109	1.79874614	5.11295251	1.34857271	1.89859993	0.81924558	2.45140269	2.43635468
12,13-DiHOME	0.43402231	0.57934598	0.25134183	0.37644227	0.56554632	0.58370129	0.6874598	0.5846151	0.47348588	0.36703148	0.59306082	0.5735239	0.57733249	0.40144543	0.61671799
9,10-DiHOME	0.26311257	0.22090212	0.20992046	0.23081707	0.37969378	0.38419046	0.34633643	0.57710293	0.31013705	0.29415134	0.28746189	0.51697985	0.45515444	0.30762094	0.32477734
15-deoxy-PGJ2	0.6427768	0.60689012	0.88098952	0.76448334	0.64016488	0.71663238	0.70670032	0.64512227	0.62491744	0.93567879	0.73492896	0.57850644	0.6558011	0.62063509	0.85475255
14(15)-EpETE	0.22852457	0.24213613	0.23923694	0.24260159	0.25610011	0.25352675	0.25383262	n.d.	0.25366639	0.26686567	0.22834504	0.25366639	0.26570866	0.25366639	0.28071661
11(12)-EpETE	0.08436677	0.08394012	0.1166757	0.16056394	0.08587002	0.05101812	0.05385666	n.d.	0.07084891	0.05237861	0.10263972	0.06866832	0.13728326	0.0674665	0.07059397
17(18)-EpETE	2.15158614	1.92911225	0.93167605	0.96982829	1.66966872	1.44699315	0.67733541	1.28778265	1.12682938	1.19326328	2.00139896	0.6286066	1.09308199	0.96125668	0.94904845
5-HEPE	0.4670141	0.47289386	0.47829585	0.46750355	0.4649204	0.46693128	0.4652697	0.4694486	0.46359326	0.48316698	0.46424091	0.46794947	0.46770209	0.46553726	0.4651784
8-HEPE	0.62354362	0.58392304	0.53031269	0.70780524	0.64726605	0.52711251	0.61937458	0.59268684	0.5916299	0.56105203	0.58487722	0.59123355	0.62173801	0.61235767	0.77913402
9-HEPE	0.42424242	0.42535968	0.45519046	0.57431075	0.54117291	0.50574276	0.4784678	0.48240964	0.42424242	0.42541554	0.42626745	0.51734291	0.45855619	n.d.	n.d.
11-HETE	0.1274768	0.12688577	0.12850397	0.12752101	0.1284985	0.12957204	0.12982702	0.13111825	0.12756639	0.12883667	0.13146075	0.12854852	0.13216303	0.12880618	0.13051398
14,15-EET	1.00837219	0.96964812	0.63445167	0.44995649	1.18464876	0.65206448	0.27555168	0.65469256	0.21494519	0.48764337	0.87728543	0.26165537	0.553969	0.52370979	1.82676953
15-HETE	0.32544435	0.17286908	0.15294005	0.29625019	n.d.	0.00594221	0.19753379	0.35702784	0.13666951	0.10796148	0.508551	0.10710506	0.15503144	0.00776212	0.3210565
5-HETE	0.22237845	0.22347061	0.22108191	0.22120108	n.d.	0.22465259	0.2304749	0.22931364	0.22581556	0.22232779	0.22465316	0.22349877	0.22581614	0.2258253	0.22815182
8(9)-EpETrE 2	5.51826696	6.52935415	7.42575645	4.15752556	20.8697736	10.5651975	4.18752232	18.5785066	2.32727698	6.01662044	9.70043652	5.78806224	5.95653632	6.64357883	4.49426226
8(9)-EpETrE	0.28091063	0.72508101	0.32372311	1.11044372	0.95816457	0.79990206	0.56161141	1.66316717	0.95662279	0.55540058	0.42639339	1.63738491	1.76439666	0.70897553	2.37921657
9-HETE	0.08329936	0.0871895	0.08714602	0.08384474	n.d.	n.d.	0.09073176	0.08349142	n.d.	n.d.	0.08676008	0.08348961	n.d.	0.08348961	0.08714783
15(S)-HETrE	0.01903915	0.06486024	0.04303001	0.03571536	0.05430614	0.02507829	0.02686573	0.02301628	0.02493994	0.03562563	0.02689951	0.04792395	0.0303417	0.05490705	0.01993037
9,10,13-TriHOME	n.d.	n.d.	n.d.	n.d.	n.d.	n.d.	n.d.	n.d.	n.d.	n.d.	n.d.	n.d.	n.d.	n.d.	n.d.
9,12,13-TriHOME	n.d.	n.d.	0.1691357	n.d.	0.00399272	n.d.	0.15483515	n.d.	n.d.	n.d.	n.d.	n.d.	n.d.	0.07000053	n.d.
11,12-DiHETE	0.6384762	0.60261642	0.59339467	0.61125971	0.63028168	0.60341279	0.61235179	0.5997189	0.63108064	0.59955288	0.59448676	0.62344124	0.6100042	0.59972668	0.60472796
14,15-DiHETE	0.68524736	0.6586276	0.63230268	0.6587088	0.64563681	0.66534915	0.65053036	0.66149992	0.64859689	0.63283189	0.64843287	0.66913535	0.64278881	0.66412683	0.63573673
17,18-DiHETE	0.62043648	0.65327362	0.59442923	0.59175536	0.62510581	0.56328169	0.59977487	0.57755928	0.58213273	0.57228613	0.63380823	0.56805808	0.5537973	0.5952792	0.62029905
5,15-DiHETE	0.74559446	0.68454722	0.73229557	0.63762076	0.61999383	0.63549609	0.65368261	0.60178109	0.6507885	0.67086362	0.74005981	0.65353397	0.68786976	0.65147924	0.69848439
6-trans-LTB4	5.96829623	5.04295256	4.99715819	5.02626946	5.15672425	4.58797373	4.73401608	4.24166971	4.47786748	4.65755661	4.50418908	4.13069472	4.76051704	4.24787183	3.64193916
LTB4	9.56962051	3.0254054	9.48678621	8.4985723	10.6483855	8.7834391	9.33536514	0.67894489	9.20865286	12.3505513	11.6270202	7.05818425	11.4579355	9.0637703	8.9565245
11,12-DiHETrE	0.73866618	0.71400538	0.69922906	0.74068832	0.78930127	0.79062076	0.83695236	0.76282922	0.74986923	0.73906128	0.74150788	0.82826952	0.83352498	0.73295115	0.91466853
14,15-DiHETrE	0.38101725	0.37562846	0.37642711	0.38939144	0.40603376	0.4624124	0.38714284	0.43419674	0.41960599	0.39091877	0.43978953	0.38462857	0.44447425	0.42763731	0.42425453
5,6-DiHETrE	0.56305297	0.54885939	0.55729352	0.70760019	0.70882137	0.65446655	0.58618543	0.78893146	0.74896248	0.63985838	0.78449002	0.73702226	0.76221961	0.5878044	0.66617197
8,9-DiHETrE	1.56500435	1.19866131	1.20185476	2.3703724	1.41537308	1.98326726	2.3631054	1.21636073	2.36159213	2.30600906	2.44933178	2.88323099	2.06958987	2.33687219	1.46084439
10(11)-EpDPE	0.49300231	0.50327917	0.47418413	0.51978808	0.47255399	0.46095481	0.4450085	0.4768785	0.44439558	0.46626172	0.46722422	0.47527027	0.46212973	0.46642398	0.44766056
13(14)-EpDPE	0.85369178	0.87461126	0.84896787	0.85545902	n.d.	n.d.	0.8580731	0.8945737	0.84095548	0.86329055	0.92152027	0.87803123	0.90031414	0.85601259	0.88546777
16(17)-EpDPE	0.73932608	0.69537523	0.69642674	0.70354252	0.73088588	0.68540591	0.71330313	0.70376727	0.72635474	0.70595458	0.69258591	0.72023429	0.71195462	0.69708093	0.73915752
16(17)-EpDPE 2	0.80763602	0.79002009	0.78008574	0.79507675	0.80124418	0.76408907	0.76510609	0.79764201	0.77729605	0.77661994	0.76380499	0.75751825	0.77349503	0.76751511	0.78449469
19(20)-EpDPE	0.65726389	0.54826042	0.67929372	0.17154879	1.13509169	1.28644408	1.05567648	0.79423545	0.58395018	0.71419994	1.28793633	0.93754146	0.7852585	0.72141106	1.23389667
7(8)-EpDPE	n.d.	n.d.	n.d.	n.d.	n.d.	4.32314928	n.d.	0.49647648	n.d.	n.d.	6.92637235	4.77422744	n.d.	n.d.	4.8544837
PGD3	2.22852963	3.66602014	2.57830646	2.1862551	2.28153051	2.18931011	2.19467823	2.56299007	2.17451892	2.20733659	2.26106094	2.427757	2.16700535	2.31737741	2.23286849
PGE3	2.80737367	4.32366569	2.53711561	2.53980064	2.53820679	2.66868691	2.47299093	3.27169757	2.51209797	2.45590506	2.40550156	2.84883072	2.37516748	2.48299034	2.484516
Resolvin	0.87143466	1.03734057	1.38402302	1.52628061	1.05162096	1.19401371	1.23465921	1.3004664	1.02133454	1.15094422	1.18682593	1.15021373	1.33656586	1.29622301	1.04021196
LXA4	1.42698118	1.69776743	0.95684784	1.24969061	1.39245557	1.13519122	1.32952235	0.84512182	1.31672396	1.05793589	1.07053475	1.32199111	0.87599023	1.04313709	0.92820721
PGD2	7.87296993	5.48437326	9.11623661	8.6047443	5.97137748	6.85022535	5.2910685	7.74321937	5.45989077	12.5380264	12.0699087	5.27015122	7.86907167	5.10428554	7.65725391
PGE2	2.58127527	2.87686741	2.54116186	2.59124076	2.66754996	2.4280984	2.42455797	2.34729875	2.50606567	2.69012132	2.40290231	2.79087067	2.41329642	3.03101432	2.81755015
8-iso-PGF1a	212.525853	206.364995	18.5740121	21.3726064	279.645631	52.0379457	43.5102125	9.25928917	110.495056	122.893915	159.225543	n.d.	4.10484515	95.593189	62.2382206
PGF2a	1.00002118	1.00981144	0.58898183	1.74624609	0.51540255	1.01559296	0.68538392	2.14923439	1.2733409	0.97009572	1.10403594	0.76277911	1.38874831	1.14306205	1.71534587
19,20-DiHDPA	0.29206528	0.37171973	0.44508005	0.48001011	0.52614895	0.44451005	0.42351582	0.31417301	0.35547226	0.39819081	0.44525978	0.40160599	0.50250394	0.39556061	0.33133999
10,11-DiHDPE	0.66653649	0.67362575	0.64791074	0.63943739	0.66293267	0.64642099	0.65158613	0.64949906	0.64816656	0.64425463	0.65894238	0.67004473	0.66634584	0.64031632	0.64435612
13,14-DiHDPE	0.54484542	0.53157894	0.53608584	0.53205169	0.54010844	0.52960704	0.52958603	0.53426207	0.53279759	0.531601	0.53363173	0.5385368	0.54663767	0.53082779	0.53373889
16,17-DiHDPE	0.5104343	0.49527747	0.49664044	0.48767488	0.48453862	0.481551	0.47375463	0.48117396	0.50314156	0.49532613	0.48836178	0.48493422	0.50824797	0.4926288	0.48520083
19,20-DiHDPE	0.42701201	0.41546659	0.37118832	0.38162619	0.512507	0.4422953	0.38994171	0.37152151	0.33641946	0.3954197	0.48253336	0.3530905	0.37390081	0.38650067	0.34576761
4,5-DiHDPE	1.91364703	1.84238392	0.33284346	1.03614905	n.d.	2.59334532	1.02683066	0.28915127	5.38131303	n.d.	1.01767238	0.27906435	2.42915446	1.28730802	2.01050281
7,8-DiHDPE	1.34823947	0.94805719	0.34219039	0.91677448	0.31875147	0.6874058	0.33366715	0.86184178	1.39210301	0.73252471	0.85598608	0.7360114	0.66651682	1.29367364	0.98144257
20-COOH-LTB4	4.10223019	4.90280359	3.13460598	3.92670271	3.54756887	2.58070109	3.27289662	5.81506552	2.381292	3.34730593	1.96274631	3.15322209	3.52382726	2.17964434	1.64794247
TXB2	1.257215	4.27380367	1.83851786	1.07953012	0.93735459	1.52162877	0.86501518	1.3504417	0.91462411	1.59565544	1.228578	0.82737346	0.57993196	1.50078846	1.94334842
6-keto-PGF1a	1.40593433	5.63979377	1.38012176	1.25756045	1.20497096	1.21316567	1.24758594	1.28597188	1.26150252	1.31077656	1.46050158	1.37141643	1.21033171	1.19540898	1.26701506

Dead cells = Gemcitabine-generated Hepa 1-6 dead cells

Supplemental Figure Legends

Supplemental Figure 1. Gene expression of PGE_2 receptors **(A)** *Ptger1* (EP1), **(B)** *Ptger2* (EP2), and **(C)** *Ptger3* (EP3) in debris-stimulated tumors (gemcitabine-generated Panc02-H7 debris ($9x10^5$ dead cells) and Panc02-H7 ($1x10^4$ living cells)) compared to control tumors (Panc02-H7 ($1x10^6$ living cells)). mRNA expression levels of target genes were analyzed by qRT-PCR and normalized by GAPDH. n = 3/group, n.s., not significant.

Supplemental Figure 2. Treatment effects of EP4 antagonists or sEH inhibitors on the cytokine storm triggered by gemcitabine-generated tumor cell debris-stimulated macrophages. **(A)** Angiogenic cytokines from conditioned medium of RAW 264.7 macrophages treated with vehicle, EC5026, TPPU, or ONO-AE3-208 at 1-10 μM (2 hours), and subsequently stimulated by gemcitabine-generated Hepa 1-6 tumor cell debris vs. macrophages alone (without debris). **(B)** Angiogenic and **(C)** inflammatory cytokines from conditioned medium of hMDMs treated with vehicle or INV-1120 at 1-20 μM (2 hours), and subsequently stimulated by gemcitabine-generated HepG2 tumor cell debris vs. macrophages alone (without debris). **(D)** Inflammatory cytokines from conditioned medium of hMDMs treated with EC5026 at 1-20 μM (2 hours), and subsequently stimulated by gemcitabine-generated HepG2 tumor cell debris vs. macrophages alone (without debris).

Supplemental Figure 3. Protein expression levels of p-IKKβ, IKKβ, p-NF-κB, and NF-κB in **(A)** RAW 264.7 murine macrophages or **(B)** hMDMs treated with TPPU (10 μM), INV-1120 (10 μM), or TPPU + INV-1120 (10 μM each) for 2 hours, and subsequently stimulated by gemcitabine-generated Hepa 1-6 or HepG2 cell debris. Western blot results were quantified by Image J. n = 3/group. * $P < 0.05$.

Supplementary Table 1. Primer sequences of target genes *Ptger1*, *Ptger2*, *Ptger3*, *Ptger4*, *Ephx2*, and GAPDH.

Supplementary Table 2. Oxylipin analysis: levels of eicosanoid metabolites in RAW 264.7 macrophage conditioned medium.

(A) Full report of concentrations (nM) of oxylipins in cell medium. LC-MS/MS–based oxylipin analysis in RAW 264.7 macrophages exposed to gemcitabine-generated Panc02-H7 dead

cells and/or INV-1120, TPPU, EC5026, or INV-1120 + TPPU. n = 3/group; n.d., not detected.

(B) Full report of concentrations (nM) of oxylipins in cell medium. LC-MS/MS–based oxylipin analysis in RAW 264.7 macrophages exposed to gemcitabine-generated Hepa 1-6 dead cells and/or INV-1120, TPPU or INV-1120 + TPPU. n = 3/group; n.d., not detected.
